# Thy1^+^ Nk Cells from Vaccinia Virus-Primed Mice Confer Protection against Vaccinia Virus Challenge in the Absence of Adaptive Lymphocytes

**DOI:** 10.1371/journal.ppat.1002141

**Published:** 2011-08-04

**Authors:** Geoffrey O. Gillard, Maytal Bivas-Benita, Avi-Hai Hovav, Lauren E. Grandpre, Michael W. Panas, Michael S. Seaman, Barton F. Haynes, Norman L. Letvin

**Affiliations:** 1 Division of Viral Pathogenesis, Beth Israel Deaconess Medical Center, Harvard Medical School, Boston, Massachusetts, United States of America; 2 Institute of Dental Sciences, Hebrew University-Hadassah School of Dental Medicine, Jerusalem, Israel; 3 Duke University School of Medicine, Duke University, Durham, North Carolina, United States of America; University of Pennsylvania, United States of America

## Abstract

While immunological memory has long been considered the province of T- and B- lymphocytes, it has recently been reported that innate cell populations are capable of mediating memory responses. We now show that an innate memory immune response is generated in mice following infection with vaccinia virus, a poxvirus for which no cognate germline-encoded receptor has been identified. This immune response results in viral clearance in the absence of classical adaptive T and B lymphocyte populations, and is mediated by a Thy1^+^ subset of natural killer (NK) cells. We demonstrate that immune protection against infection from a lethal dose of virus can be adoptively transferred with memory hepatic Thy1^+^ NK cells that were primed with live virus. Our results also indicate that, like classical immunological memory, stronger innate memory responses form in response to priming with live virus than a highly attenuated vector. These results demonstrate that a defined innate memory cell population alone can provide host protection against a lethal systemic infection through viral clearance.

## Author Summary

Immunological memory is a hallmark of adaptive immunity and provides the basis for our ability to become ‘immune’ to pathogens to which we have previously been exposed, and provides the basis for vaccination. For decades, the paradigm held that only the classical adaptive lymphocytes were capable of forming and maintaining protective immunological memory. Recently, several papers have shown the capacity of an innate cell population, a subset of natural killer (NK) cells, to exhibit certain aspects of immunological memory. Here we show that innate memory forms in response to infection with vaccinia virus and resides in a discrete subset of NK cells. We further demonstrate that this innate memory provides significant host protection against a subsequent systemic infection with a lethal dose of vaccinia virus, in some cases resulting in the complete clearance of detectable virus. We also demonstrate that priming with live, replicating virus stimulates innate memory more robustly than a highly attenuated vector. These findings shed new light on this emergent area of immunology, and hold significant implications for harnessing innate memory as part of creating novel vaccination strategies.

## Introduction

Immunological memory allows the immune system to provide enhanced host protection upon secondary exposure to an infectious pathogen. Memory has long been considered the sole province of adaptive lymphocytes. Lymphocytes recognize pathogens via unique somatically-rearranged antigen receptors, expand clonally upon activation, and eventually give rise to a population of long-lived progeny. These progeny cells maintain their antigenic specificity and exhibit enhanced functional activity upon secondary exposure to a priming pathogen.

Recent studies have suggested that a reconsideration of the classical paradigm of immunological memory is warranted. These studies have shown that innate cell populations have the capacity to generate enhanced responses upon secondary exposure to the priming immunogen[1], [2], [3]. O'Leary, et al., demonstrated that NK cell-mediated delayed-type hypersensitivity (DTH) responses[1] can be generated upon secondary exposure to sensitizing compounds. Further, they showed that these compound-specific DTH responses were mediated by a liver-resident NK cell population expressing the Thy1 (CD90) molecule. Recently, Sun, et al. demonstrated that an innate memory response forms to MCMV and is mediated by a population of NK cells expressing Ly49H. In mice containing the B6 haplotype NK complex, NK cells expressing the germline-encoded NK receptor Ly49H—an activating NK receptor identified that is capable of specifically recognizing a virally-encoded product (the MCMV protein m157)[4], [5]—are the predominant contributors to the innate response to a primary MCMV infection[2], [6], [7], [8], [9], [10], [11]. Sun et al., made use of the cognate recognition of m157 by Ly49H^+^ to establish the specificity of the enhanced host protection provided by memory Ly49H^+^ NK cells upon re-exposure to MCMV[2].

Recently, a report from the von Andrian laboratory [3] showed that CXCR6^+^ NK cells primed with virus-like particles (VLPs) expressing viral transgenes were capable of mediating antigen-specific contact hypersensitivity (CH) in response to antigens not known to be recognized by germline-encoded receptors. These memory NK cells were also capable of providing partial host protection from infection with live virus expressing the priming antigens, as measured by a delay in mortality upon lethal viral challenge and the capacity to resolve localized, lower dose viral infection. Further, they demonstrated that signaling through CXCR6, a chemokine receptor that binds to CXCL16 (primarily expressed by hepatic sinusoidal epithelium), is critical for the maintenance of this hepatic NK memory population of cells.

The present studies were initiated to determine whether innate memory could contribute to the control of viral pathogens for which no cognate germline-encoded receptor has been identified. Furthermore, we wanted to assess whether innate memory cells alone can provide protection against a viral challenge in the absence of classical adaptive immunity. In this series of experiments, we used live vaccinia virus to prime and challenge the memory NK cell population and demonstrate that memory NK cells alone are capable of providing sterilizing protection against a systemic challenge with a fatal dose of the priming pathogen. This protection was mediated by a Thy1^+^ subset of hepatic NK cells primed with live vaccinia virus; hepatic Thy1^+^ NK cells primed with a highly attenuated strain of vaccinia (Modified Vaccinia Ankara (MVA)) were unable to confer this protection upon adoptive transfer into naïve, immunodeficient hosts. This suggests that innate memory, like classical adaptive memory, is primed more efficiently with live, replication-competent organisms. We demonstrate that innate memory to vaccinia virus is extremely durable, as the memory NK cells that mediated clearance of the lethal pathogen were active greater than 6 months after priming with the live pathogen. We also show that on a cellular level, enhanced activation of hepatic NK cells from virally primed mice can be demonstrated not just in response to the pathogen, but in response to more generalized stimuli.

## Results

We chose vaccinia virus (VV) as a pathogen for these studies because immunocompetent mice resolve vaccinia virus infection through a combination of humoral and cell-mediated adaptive immune responses[12]. Moreover, the virus is highly pathogenic in naïve mice that lack classical adaptive immune function. By infecting mice with a recombinant vaccinia virus expressing the transgene firefly luciferase (rVV-luc), we were able to monitor the infection *in vivo* by periodically injecting luciferin-D (the luminescent substrate for firefly luciferase) into the mice and using the IVIS imaging system to monitor viral burdens and virus localization.

First, we infected mice in which the IgHμ constant region has been disrupted (the μMT mouse; IgH^ko^)[13] which results in a consequent failure to develop mature B cells. The genetic block in B cell development is extremely stringent when in C57Bl/6 (B6) mice[13], [14] (data not shown). We used the IgH^ko^ B6 mice for these studies to eliminate the possibility that a humoral immune response might develop during a primary infection with vaccinia virus that could confer protection against a secondary exposure to virus. We also reasoned that T lymphocytes present in these mice would clear a primary vaccinia virus infection efficiently; RAG1^ko^ mice (mice that lack both T and B cells) succumb to primary infection with vaccinia virus at very low doses. In using the IgH^ko^ B6 mouse model, we could prime mice in the presence of T cells, thereby allowing the IgH^ko^ mice to survive the primary infection, and then eliminate cell subpopulations *in vivo* by administration of monoclonal antibodies prior to secondary challenge. Antibody-mediated depletion of T cells could be maintained indefinitely in the IgH^ko^ mice by repeated administration of depleting antibodies since these mice cannot generate anti-Ig antibody responses that might interfere with the infused antibodies.

### A non-T-, non-B- lymphocyte population provides host protection upon secondary exposure to vaccinia virus

**Figure 1:**
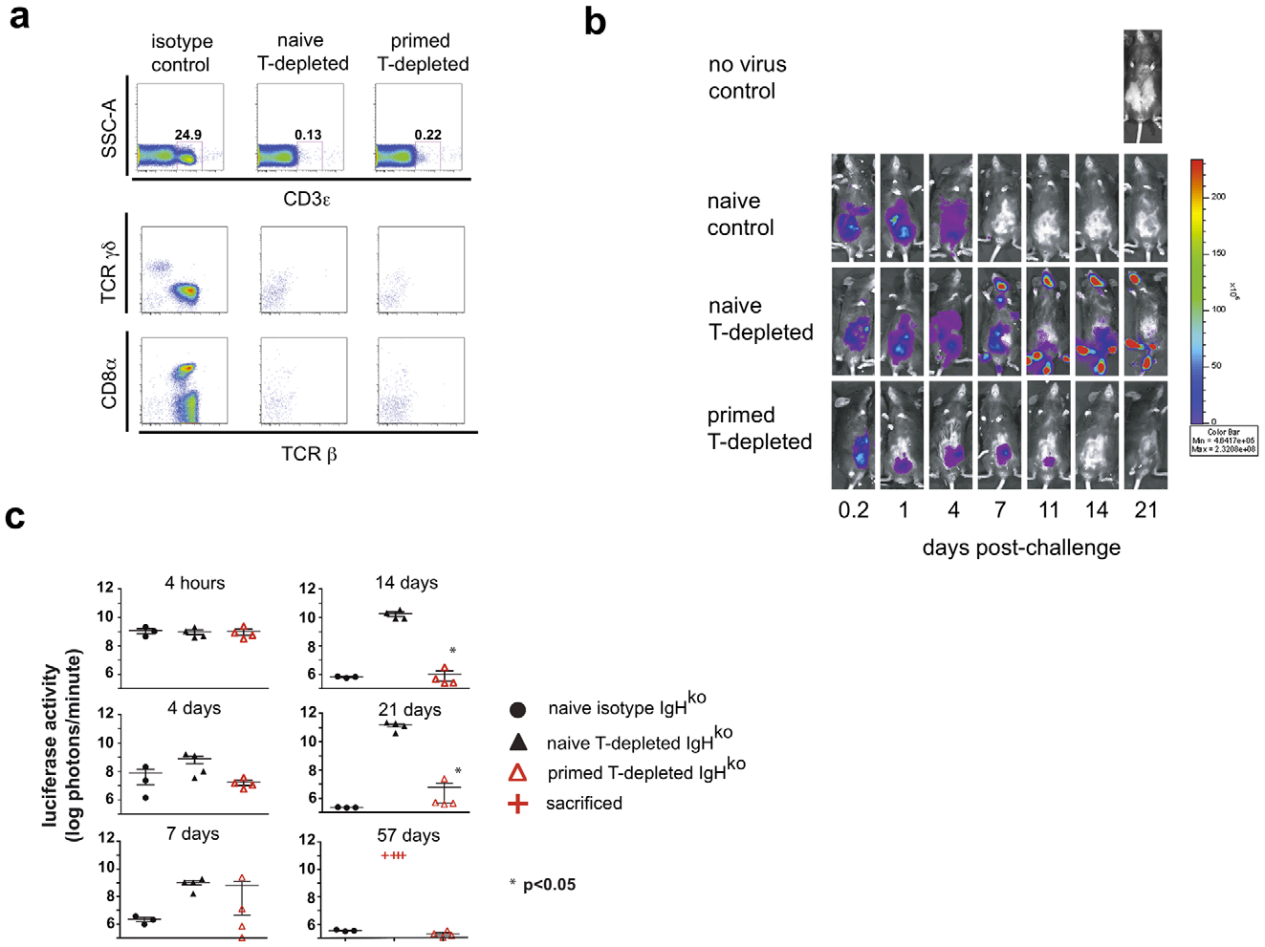
A primed innate cell population provides protection against a challenge with recombinant vaccinia virus. Six months after intraperitoneal (ip) inoculation with vaccinia virus (primed) or PBS (naïve), IgH^ko^ mice were treated with T cell-depleting or isotype control antibody mixtures and 10 days later were challenged with 2×10^6^ pfu rVV-luc intraperitoneally (*ip*). Isotype control or depleting monoclonal antibodies were administered ip every 2 weeks starting a week to ten days prior to viral challenge. (a) Flow cytometric plots of CD3 staining of peripheral blood cells from naïve control, naïve T-depleted, or primed T-depleted mice that fall within the lymphocyte gate are shown, including the percentage of total cells within the CD3^+^ gate at 1 week post-challenge. The TCRβ, TCRγδ, and CD8α staining profiles of the cells that fall within the CD3^+^ gate are also shown. (**b**) IVIS images of representative mice from the naïve control, naïve T cell-depleted, and primed T cell-depleted groups following the challenge with rVV-luc are shown. (**c**) The measurements of rVV-luc viral loads in each group over time are shown with relative viral loads based on the number of photons emitted normalized for a one minute exposure. The measurement for each individual mouse is plotted, and the center line indicates the mean with error bars representing the SEM. Statistical significance between T-depleted groups was assessed by t-tests using Mann-Whitney analysis and is indicated by the asterisk (p<0.05). Results are representative of 4 independent experiments.

IgH^ko^ mice received an inoculation of either PBS (control naïve IgH^ko^), or 1×10^7^ plaque forming units (pfu) of rVV-luc intraperitoneally (ip) (primed IgH^ko)^). We then rested the mice for a minimum of six months after clearance of the rVV-luc (as determined by IVIS imaging; **Fig. S1**) to ensure that any effects we observed during a secondary challenge would reflect the influence of a durable, long-lived memory immune response rather than the activity of residual effector cell populations that may have persisted following clearance of the virus. Ten days prior to a secondary vaccinia virus challenge we began administering isotype control antibodies or a cocktail of T cell- depleting monoclonal antibodies (clones GK1.5 (αCD4)[15], H57–597 (αTCRß)[16], and UC7-13D5 (αTCRγδ)[17]) ip to groups of naïve and primed animals. We did not include a CD8α-depleting monoclonal antibody in this cocktail because we did not want the depletion of CD8α^+^ innate populations of cells to potentially confound the outcome of the experiments. The monoclonal antibody mixtures were administered every two weeks throughout the life of the experiment to maintain T cell depletion. The efficiency of T cell depletion was monitored by flow cytometric analysis of peripheral blood stained with a panel of monoclonal antibodies, including antibodies specific for CD3, TCRβ, TCRγδ, and NK1.1. Representative flow cytometry plots from control and T cell-depleted mice 1 week post-challenge showed that the efficiency of T cell depletion was extremely high, greater than 99% as measured by percentage of CD3^+^ within the lymphocyte gate (**Fig. 1a**). Very few of those cells that fall within the CD3^+^ gate in the depleted mice showed staining for TCRβ, TCRγδ, or CD8α; there are no masking antibodies to CD3 or CD8α present in the cocktail. Moreover, pilot studies performed in control B6 mice showed that the extent of T cell depletion in the peripheral blood following administration of the depleting antibodies reflected the T cell depletion in spleen and lymph nodes (data not shown).

In repeated experiments, we observed that both primed (data not shown) and naïve IgH^ko^ mice treated with isotype control antibodies were capable of clearing vaccinia virus infection. However, in the T cell-depleted groups of mice, only the primed mice (primed, T-depleted IgH^ko^) were capable of resolving the infection (**Fig 1** **, b** and **c**). Representative IVIS images show the kinetics of vaccinia virus clearance in each of the groups of mice (**Fig. 1b**). The luminescence measurements on all mice demonstrated that while naïve, T-depleted IgH^ko^ were incapable of controlling vaccinia virus after challenge, the primed T-depleted IgH^ko^ mice resolved the infection **(** **Fig. 1c** **)**. The clearance of vaccinia virus infection occurred within 2–3 weeks in primed, T cell-depleted mice (**Fig. 1**, **b** and **c**), more slowly than virus clearance occurred in primed, control IgH^ko^ mice (clearance in 1 week-data not shown) or in naïve, control IgH^ko^ mice (clearance in 10–14 days; **Fig. 1**, **b** and **c**). Once the primed, T cell-depleted mice had resolved the vaccinia virus infection, there was no evidence of a later recrudescence of viral replication. In mice that succumbed to vaccinia (naïve, T cell-depleted IgH^ko^), we observed spread of vaccinia virus from the peritoneal cavity into hotspots located on the tails, feet, and mouths of infected mice. This distribution of vaccinia virus was consistent with previous reports[12] and was likely the result of transfer of infection to satellite sites via saliva. Together, these data suggest that the long-term resolution of vaccinia virus infections can occur in the absence of classical adaptive immune effector mechanisms.

### Thy1 (CD90) expression identifies the non-T, non-B cell population that provides protection upon secondary exposure to vaccinia virus

**Figure 2:**
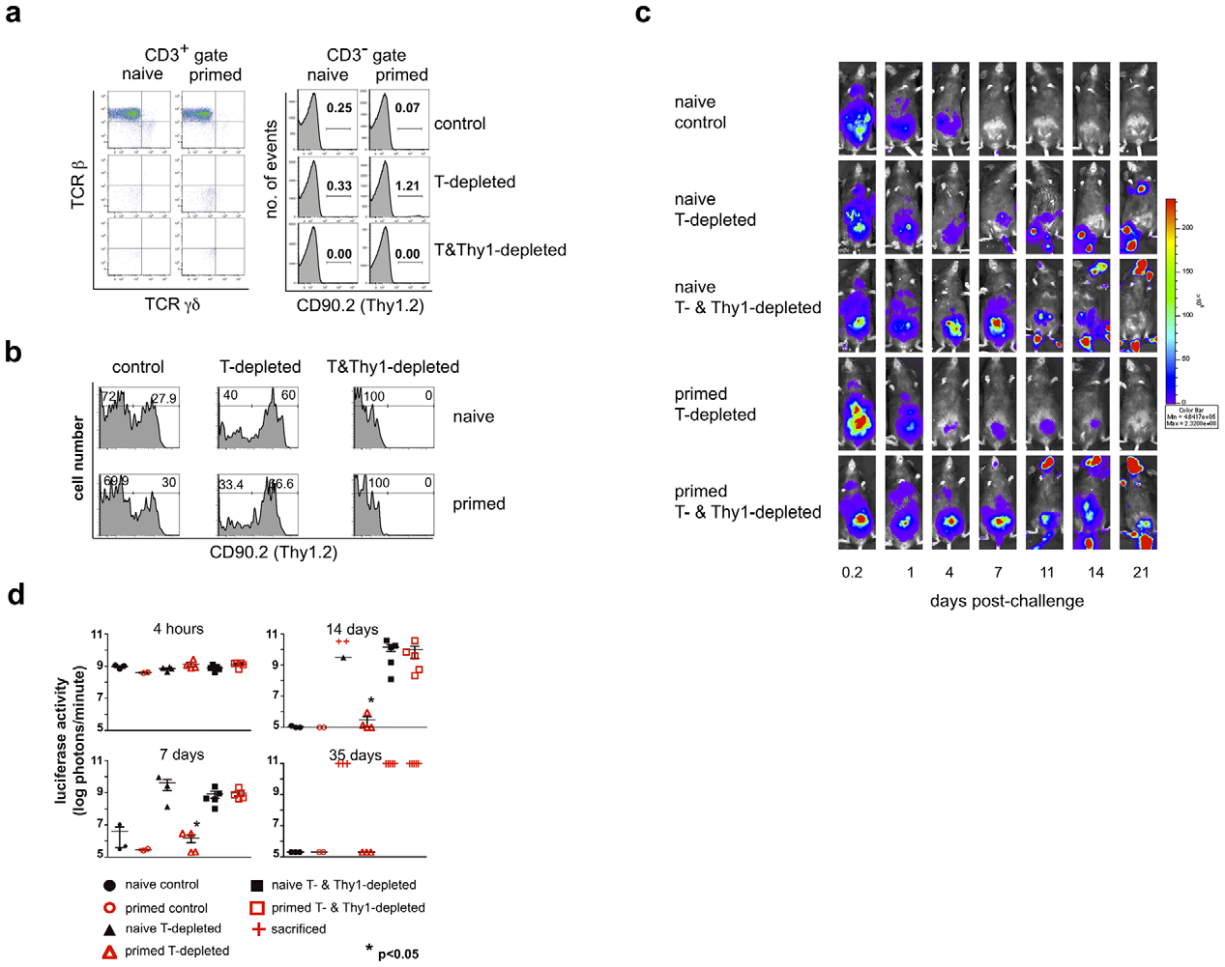
A Thy1^+^ cell population mediates innate protection against a secondary challenge with vaccinia virus. Eight months after the initial administration of vaccinia virus (primed) or PBS (naïve), IgH^ko^ mice were treated with either isotype control or depleting monoclonal antibodies, then challenged ten days later with 2×10^6^ pfu rVV-luc. Isotype control and depleting monoclonal antibodies were administered ip every 2 weeks beginning one week to ten days prior to viral challenge. (**a**) Representative flow cytometric plots of staining of all cells that fall within the CD3^+^ and CD3^−^ lymphocyte gates from peripheral blood 2 weeks after the secondary challenge are shown. (b) Histograms of CD90.2 expression by the NK cell population (gated on CD3^−^NK1.1^+^ cells) isolated from peripheral blood of each of the indicated groups are shown. (**c**) IVIS images of representative IgH^ko^ mice from naïve isotype control, naïve and primed T cell-depleted, and naïve and primed T- and Thy1(CD90.2)^+^- cell depleted groups are shown at various time points following secondary challenge with rVV-luc. (**d**) The measurements of rVV-luc viral loads in each group over time are shown, with relative viral loads based on the number of photons emitted normalized for a one minute exposure. The measurement for each individual mouse is plotted, and the mean is indicated by the centerline with error bars representing the SEM. Statistical significance between T-depleted and T&Thy1-depleted groups was assessed by t-tests using Mann-Whitney analysis and is indicated by the asterisk (p<0.05). Results are representative of 4 independent experiments.

We next sought to identify the cell population(s) that mediated this non-T and non-B cell protective memory response *in vivo*. Since Thy1^+^ lymphokine-activated killer (LAK) cells have been implicated in protection against infection with vaccinia virus[18], [19], [20], [21], [22], and O'Leary, et al., have shown that the multiple compound-specific DTH responses they observed are mediated by a liver-resident Thy1^+^ NK cell population[1], we hypothesized that Thy1^+^ NK cells may be the effector population providing protection against secondary vaccinia virus infection. To explore this possibility, we inoculated groups of IgH^ko^ mice with either 1×10^7^ pfu rVVluc (primed IgH^ko^) or PBS (naïve IgH^ko^) ip. Eight months later, these mice were treated either with isotype control antibodies, the T cell-depleting antibody cocktail (300 μg each of clones GK1.5, H57–597, and UC7-13D5), or a T- and Thy1-depleting antibody cocktail (300 μg each of clones GK1.5, H57–597, UC7-13D5, and the Thy1.2 (CD90.2)-specific clone 30H12[23]); the monoclonal antibody mixtures were administered every 2 weeks throughout the course of the experiment to maintain T and Thy1^+^ cell depletion. One week after initiating monoclonal antibody administration, all groups of mice were challenged ip with 1×10^6^ pfu rVV-luc. Representative flow cytometric plots of peripheral blood cells from groups of naïve and primed mice 2 weeks after challenge demonstrate that the depletion of the target cell populations by monoclonal antibody administration was extremely efficient (>99% T cell depletion in all groups at 2 weeks post-challenge; **Fig. 2a**). Depletion of T cells had no significant impact on the number of NK cells present in the peripheral blood. We did observe a significant shift in the representation of Thy1 (CD90)^+^ NK cells in mice that had been depleted of T cells. Nevertheless, the proportion of Thy1^+^ NK cells was comparable in the naïve and primed groups (**Fig. 2b**), suggesting that differences in clearance and survival between the naïve and primed T-depleted groups is not a consequence of differential effects of T cell depletion on the NK cell populations. As anticipated, the addition of the Thy1.2 (CD90.2)-depleting monoclonal antibody 30H12 in the T&Thy1-depleting cocktail efficiently eliminated all Thy1^+^ NK cells in IgH^ko^ recipients (**Fig. 2b**). Both the naïve and primed IgH^ko^ control (T cell-competent) groups of mice cleared the infection with kinetics consistent with primary (naïve IgH^ko^) and secondary (primed IgH^ko^) adaptive immune responses, respectively (**Fig. 2** **, c** and **d**). As observed in the initial series of experiments, the naïve, T-cell-depleted IgH^ko^ mice were unable to control the challenge infection while the primed, T cell-depleted IgH^ko^ mice resolved the vaccinia virus infection within 2–3 weeks. However, depletion of both T cells and Thy1^+^ non-T cells abrogated the protection that was evident in the primed T-cell-depleted IgH^ko^ group (**Fig. 2c and d**). The naïve T- and Thy1-depleted mice also succumbed to infection. These data suggested that the cell population(s) mediating protection against secondary vaccinia virus challenge was a Thy1^+^, non-T, non-B cell population.

To examine the comparability of data generated using the luciferase/IVIS system and data generated using a traditional strategy for measuring vaccinia virus infection *in vivo*, we quantified vaccinia virus titers in ovaries of primed T-depleted and primed T- and Thy1-depleted groups of mice. The results established that the clearance of luciferase activity in primed T-depleted animals as visualized using the IVIS technology correlated with an inability to detect vaccinia virus in ovaries of these animals (**Fig. S2** and data not shown). This observation indicates that viral clearance as measured by IVIS is consistent with traditional methods of measuring viral clearance.

### NK cells from pathogen-primed mice retain enhanced effector capacity long after clearance

**Figure 3:**
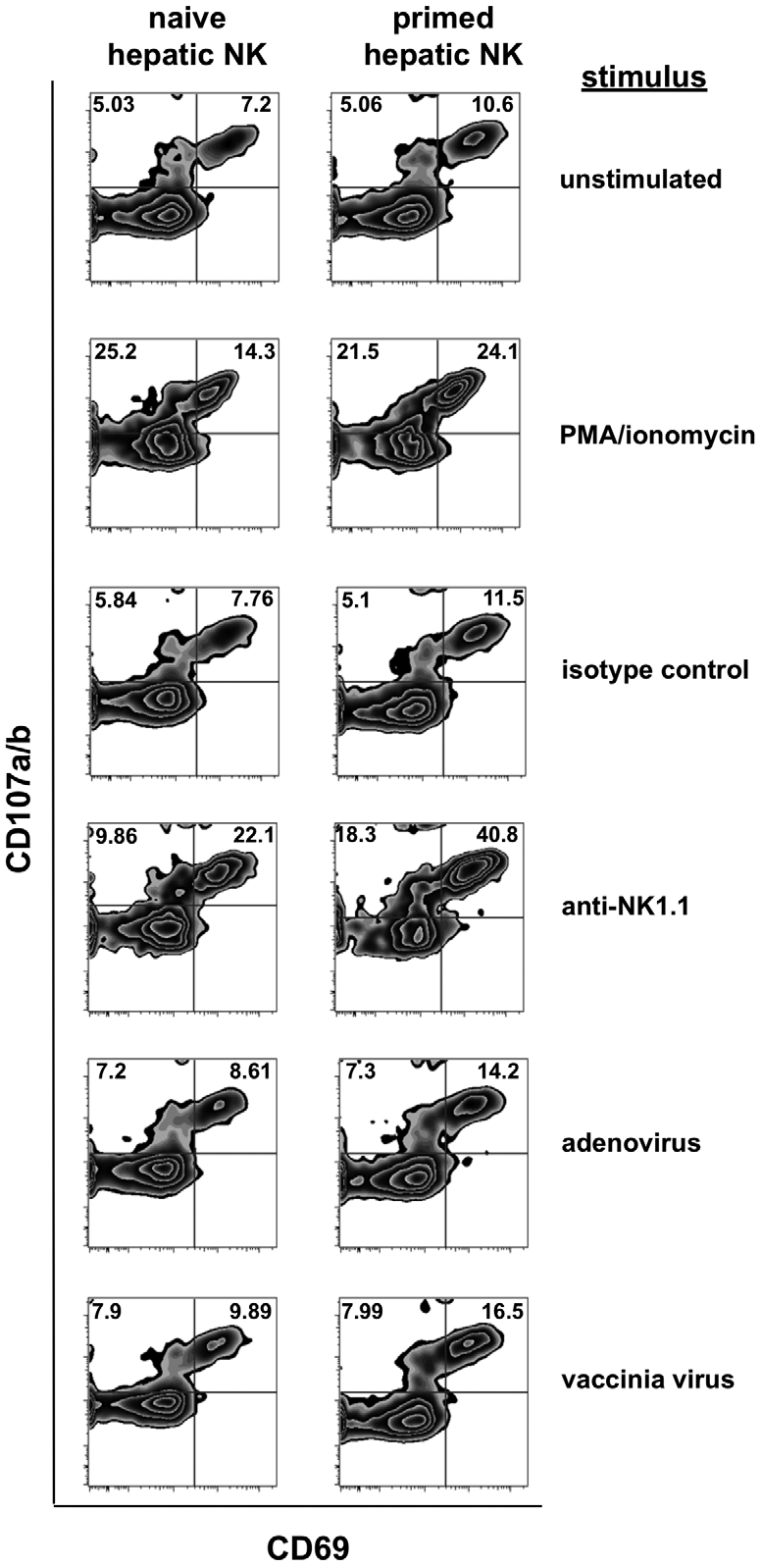
Intracellular cytokine staining of previously primed NK cells demonstrates enhanced activation following in vitro stimulation. Lymphocytes and NK cells isolated from collagenase-dissociated liver preparations from naïve and vaccinia virus-infected mice were cultured *in vitro* with the indicated stimuli for 6 hours prior to evaluation for NK cell activation (expression of CD69) and degranulation (expression of CD107) in response to the stimulation. Stimuli include plate-bound isotype control Ig, the anti-NK1.1 monoclonal antibody PK-136, formaldehyde-fixed adenovirus (2×10^6^ vp), and formaldehyde-fixed vaccinia virus (2×10^6^ pfu). Plots shown are gated on NK cells (NK1.1^+^CD3^−^ or DX5^+^CD3^−^).

One of the fundamental characteristics of a classical memory response is that memory cells exhibit enhanced activation upon secondary stimulation. Therefore, we wanted to determine if functional differences existed between NK cells from naïve and vaccinia virus-primed mice. To do so, we performed *in vitro* activation assays using isolated liver mononuclear cell preparations. We pooled liver mononuclear cells from groups of 8–12 age-matched naïve mice and mice that had been VV-primed 6 months earlier to ensure that we could isolate sufficient numbers of cells for stimulation and analysis. These cell preparations were isolated from enzymatically-dissociated livers via percoll gradient separation and 5×10^5^ cells/well were aliquoted into 24 well plates. The cells were then incubated for 6 hours in the presence of fluorescently-labeled anti-CD107a/b antibodies in wells containing either no stimulus, PMA and ionomycin, 200 micrograms of plate-bound isotype control antibody (clone CI.8), 200 micrograms of plate-bound anti-NK1.1 antibody (clone PK-136), or either 2×10^6^ pfu of vaccinia virus or 2×10^6^ viral particles (vp) of recombinant adenovirus that had been treated with 2% paraformaldehyde. The recovered cells were washed, stained for CD69 expression, and fixed prior to flow cytometric analysis. In each condition, analysis on gated NK cells (NK1.1^+^CD3^−^) showed that the NK cells from vaccinia virus-primed animals showed higher activation, as determined by CD69 (activation) and CD107a/b (degranulation) staining (**Fig. 3**). Primed NK cells showed significantly higher levels of activation and degranulation than naïve NK cells when stimulated with PMA and ionomycin (24.1% CD69^+^CD107^+^ for primed NK vs. 14.3% for unprimed; specific activation above unstimulated 13.5% for primed vs. 7.1% for unprimed NK), and plate-bound anti-NK1.1 antibody (40.8% primed vs. 22.1% unprimed; specific activation above isotype-stimulated 29.3% primed vs. 14.3% unprimed). This activation occurred in response to the indicated stimuli, as activation was well above control levels, and by stimuli that were not dependent on a presenting cell population or the presence of vaccinia virus antigens. The cells also showed an enhanced response to fixed, plate-bound vaccinia virus when compared to the unstimulated control cells (16.5% primed vs. 9.9% unprimed; specific activation above unstimulated 5.9% primed vs. 2.7% unprimed), although the response to vaccinia virus was only marginally higher than the response to an unrelated virus (16.5% primed vs. 9.9% unprimed responded to vaccinia virus, while responses to adenovirus were 14.2% primed vs. 8.6 unprimed; vaccinia-induced activation relative to adenovirus-induced activation 2.3% primed vs. 1.3% unprimed). These results showed that, as with the non-specific stimuli, primed NK cells exhibited significantly enhanced activation relative to unprimed NK cells in response to direct exposure to viral particles. However, the relatively small increases in activation seen in both primed and unprimed cells in response to vaccinia virus as compared to an unrelated virus (adenovirus) suggest that direct and specific recognition of vaccinia virus antigens plays a limited role in stimulating both vaccinia –primed and –unprimed NK cells. Collectively, these results show that NK cells from vaccinia virus-primed mice were more responsive than NK cells from naïve mice to a wide range of activating stimuli.

### A Thy1^+^ subset of NK cells dominates the NK response during primary infection with vaccinia virus

**Figure 4:**
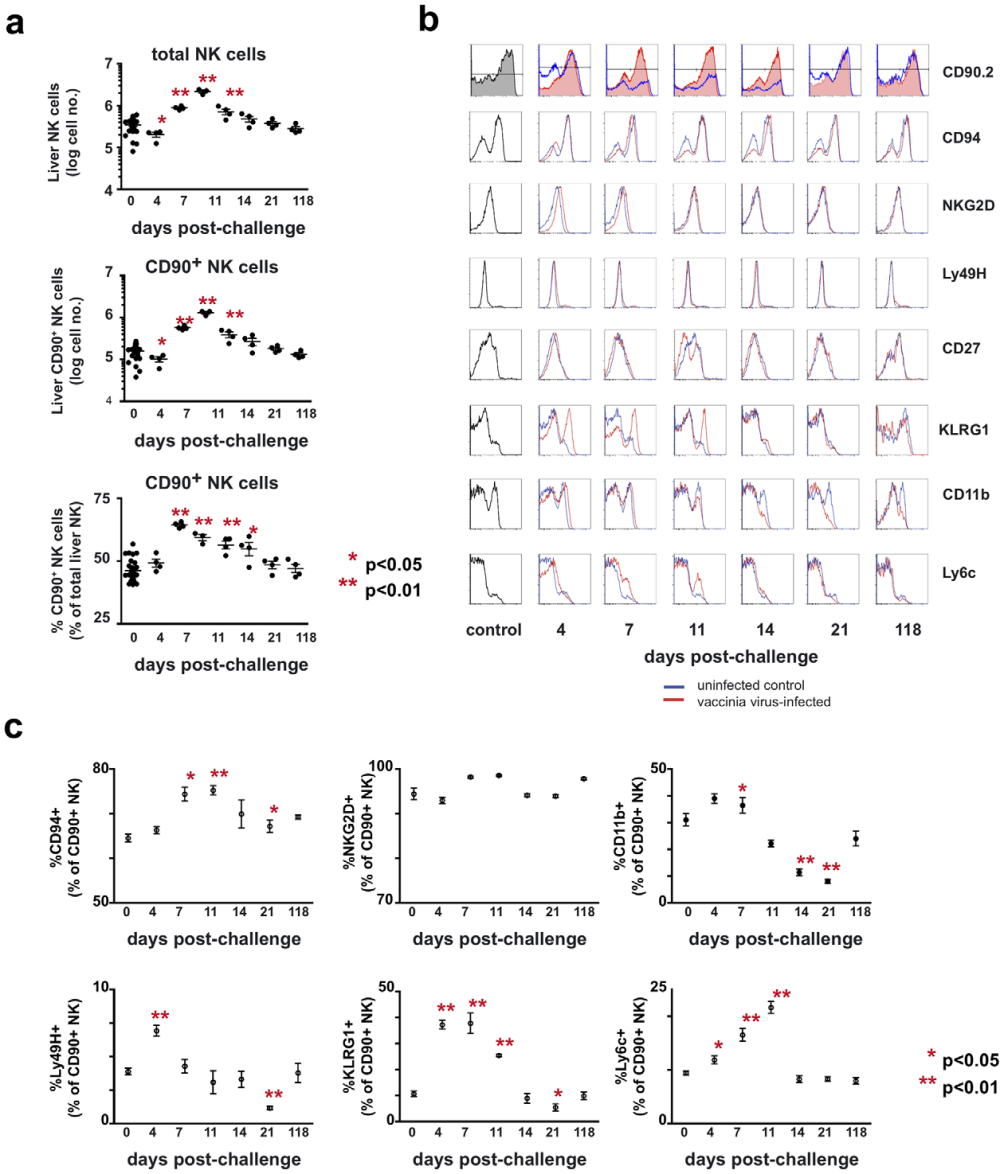
Kinetic and phenotypic analysis of liver NK cell responses following primary vaccinia virus infection reveals the preferential expansion of a Thy1^+^ population. Mice were administered either PBS (control) or 1×10^7^ pfu of rVV (challenged) ip. At various time points following challenge, cells were isolated from the enzymatically-dissociated livers of control and challenged mice. (**a**) The absolute number of NK cells (CD3^−^NK1.1^+^), the absolute number of Thy1^+^ NK cells (CD3^−^ NK1.1^+^ Thy1^+^), and the percentage of total NK cells that were Thy1^+^ are shown. Each data point represents an individual mouse, with the mean indicated by the horizontal bar and error bars representing the SEM. Absolute liver NK cell numbers were calculated by multiplying the total number of viable cells within the lymphocyte gate of each sample (as determined by analysis on a Guava easyCyte instrument) by the percentage of those cells determined to be CD3^−^NK1.1^+^ by flow cytometric analysis. The representation of Thy1^+^ cells within the total NK cell population, both as a percentage and an absolute number, was also determined based on flow cytometric analysis and the cell counts obtained by Guava analysis. Statistical analyses were performed using t tests and Mann-Whitney analysis comparing the various time points to that of the naïve control group. Statistically significant differences between virally-infected groups post-challenge and the uninfected control group are indicated by one (p<0.05) or two (p<0.01) asterisks. (**b**) Further phenotypic characterization of Thy1^+^ liver NK cells following vaccinia virus infection is shown in a series of histograms. The red traces represent cells from challenged mice and the blue traces represent cells from control mice isolated at the same time point and stained with the same antibody cocktail. (**c**) Quantitative analyses of the phenotypic profiles of Thy1(CD90)^+^ NK cells (cells within the CD3^−^NK1.1^+^CD90.2^+^ gate) stained with antibodies to the indicated molecules as determined by flow cytometric analysis. Statistical analyses were performed using t tests and Mann-Whitney analysis comparing the various time points to that of the naïve control group. Statistically significant differences between virally-infected groups post-challenge and the uninfected control group are indicated by one (p<0.05) or two (p<0.01) asterisks.

We next characterized the dynamics of NK cell populations throughout the course of vaccinia virus infection in unmanipulated, wild type mice. B6 mice were administered PBS (control) or 1×10^7^ pfu of rVV (challenged) ip. At various time points after infection, groups of control and challenged mice were sacrificed, and cells from the peripheral blood, spleen, and liver were isolated, counted, and analyzed by monoclonal antibody staining and flow cytometry. A rapid expansion was seen in the absolute number of NK cells in the spleen (data not shown) and liver (**Fig. 4a** **)** during the first week following challenge, followed by a contraction of this population as the infection resolved. By 7 days following infection, the absolute number of liver NK cells (CD3^−^NK1.1^+^) had increased 5-fold. There was a preferential expansion of the Thy1^+^ NK cell population in the liver of infected mice, with the representation of Thy1^+^ cells increasing in the liver both as a percentage of total NK cells (from 46% to 64% of total liver NK cells by day 4 post-challenge) and in their absolute number (a 10-fold increase over baseline by 7 days after infection). A preferential expansion of splenic Thy1^+^ NK cells was also observed, albeit to a lesser degree (approximately 2–fold expansion in the total number of splenic NK cells, and a 5-fold expansion in the number of Thy1^+^ splenic NK cells; the percentage of total splenic NK cells that were Thy1^+^ rose from a baseline of 32% to a peak of 57% by 4 days post-infection; data not shown).

We also undertook a detailed phenotypic analysis of the liver NK cells following vaccinia virus infection. These cells were stained with antibodies specific for NK-associated cell surface molecules, including CD94, NKG2D, CD43, Ly49H, Ly6C, KLRG1, CD27, CD11b, and Thy1 (**Fig. 4** **, b and c**). During the first week after vaccinia virus challenge, the absolute number of all NK subsets we analyzed had increased. However, we were able to identify several NK cell subsets that preferentially increased in number and percentage: the total Thy1^+^ NK cell population, as described above, and a subpopulation of Thy1^+^ NK cells that were also KLRG1^hi^. Most of these cells were phenotypically NKG2D^bright^Ly6C^hi^CD27^lo^CD11b^hi^CD43^hi^ (**Fig. 4** **, b and c,** and data not shown). The representation of this KLRG1^hi^ population rose from approximately 5% to a peak of 20–25% of the liver Thy1^+^ NK cell population by days 4–7 after infection, and then contracted to baseline levels by 21 days after challenge (**Fig. 4b and c**). However, by 118 days after challenge there were no phenotypically discrete subsets within the Thy1^+^ NK cell subset that we could identify as potential memory populations.

### Expansion of NK cells in liver results from *in situ* proliferation

**Figure 5:**
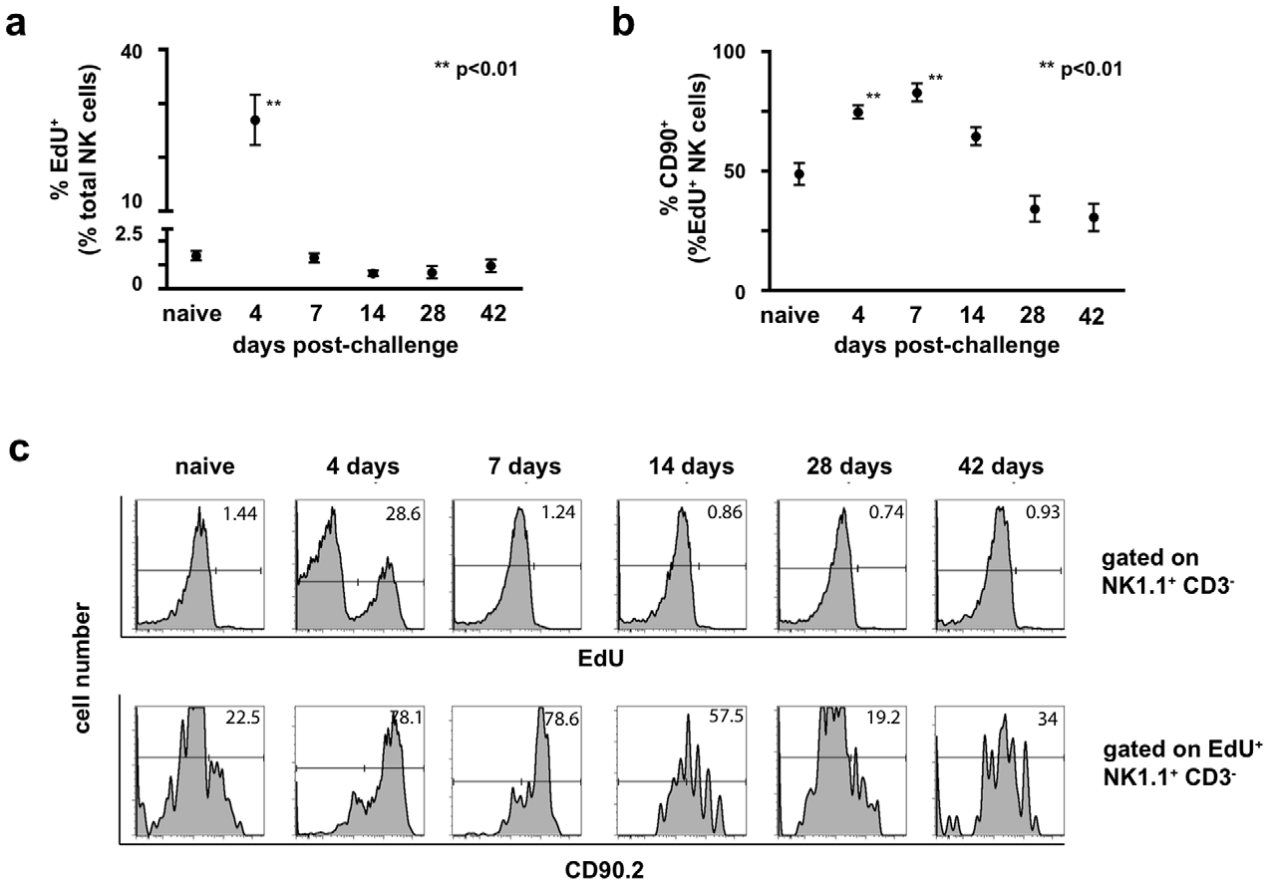
Expansion of the NK cell population post-challenge is driven by proliferation *in situ*. Groups of B6 mice were either infected with 1×10^7^ pfu rVV *ip* or given a control injection of PBS. The thymidine analog 5-ethynyl-2′-deoxyuridine (EdU) was administered *ip* 12 hours prior to sacrifice (250 μg EdU/animal). Cell suspensions isolated from liver were stained with monoclonal antibodies to CD3, NK1.1, and Thy1 (CD90.2), fixed, permeabilized, and treated with the Click-IT EdU staining buffer with Alexa 647 azide to detect EdU incorporation. (a) The percentage of liver NK cells (CD3^−^NK1.1^+^) that incorporated EdU is plotted over time. Statistical analyses were performed using t tests and Mann-Whitney analysis comparing the various time points to that of the naïve control group. Statistically significant differences between virally-infected groups post-challenge and the uninfected control group are indicated by one (p<0.05) or two (p<0.01) asterisks. (b) The percentage of EdU^+^ liver NK cells expressing Thy1 (CD90.2) is plotted over time. Statistical analyses were performed using t tests and Mann-Whitney analysis comparing the various time points to that of the naïve control group. Statistically significant differences between virally-infected groups post-challenge and the uninfected control group are indicated by one (p<0.05) or two (p<0.01) asterisks. (c) Representative histograms measuring EdU incorporation by total liver NK cells is shown in the top panel, while representative histograms measuring Thy1 (CD90.2) expression by cells within the corresponding EdU^+^ gate are represented in the lower panel. These results are representative of two independent experimental replicates.

We wanted to determine whether the expansion of Thy1^+^ NK cells observed in the liver and spleen during primary infection were a consequence of proliferation or an alteration in NK cell trafficking. Groups of B6 mice were infected with 1×10^7^ pfu rVV *ip* , and at various time points administered the thymidine analogue ethynyl deoxyuridine (EdU) 12 hours prior to sacrifice. We designed the experiment to allow the assessment of the proliferative activity within the NK cell population at various time points rather than following continuous EdU administration. As shown in **Fig. 5**, extensive proliferation within the NK cell population occurred early in infection (25% of total NK cells proliferating between days 3–4 post-challenge), and returned to baseline levels by 7 days post-challenge (**Fig. 5a**). The preferential expansion of Thy1^+^ NK cells was seen at both day 4 and 7 post-challenge (where the percentage of EdU^+^ NK cells that were also Thy1^+^ reached levels of 74.6% and 82.9% respectively, compared to 49% in naïve controls; **Fig. 5b**). These results are consistent with the population dynamics observed in the liver NK cell population (**Fig. 4a**), and suggest that the large increases in NK cell number and Thy1^+^ NK cell percentage in liver are driven by *in situ* proliferation early in infection. In support of the mechanism of *in situ* proliferation, plaque assays performed on *de novo* infected animals showed that low levels of vaccinia virus could be recovered from the livers of animals receiving vaccinia virus *ip* beginning at 4 hours post-infection and through day 4 post-infection (ranging from 4×10^2^–5×10^4^ pfu; data not shown). This finding established that pathogen is also present *in situ* and might directly stimulate the expansion of Thy1^+^ NK cells.

### Adoptive transfer of vaccinia-primed hepatic Thy1^+^ NK cells confers protection on naïve immunodeficient hosts

**Figure 6:**
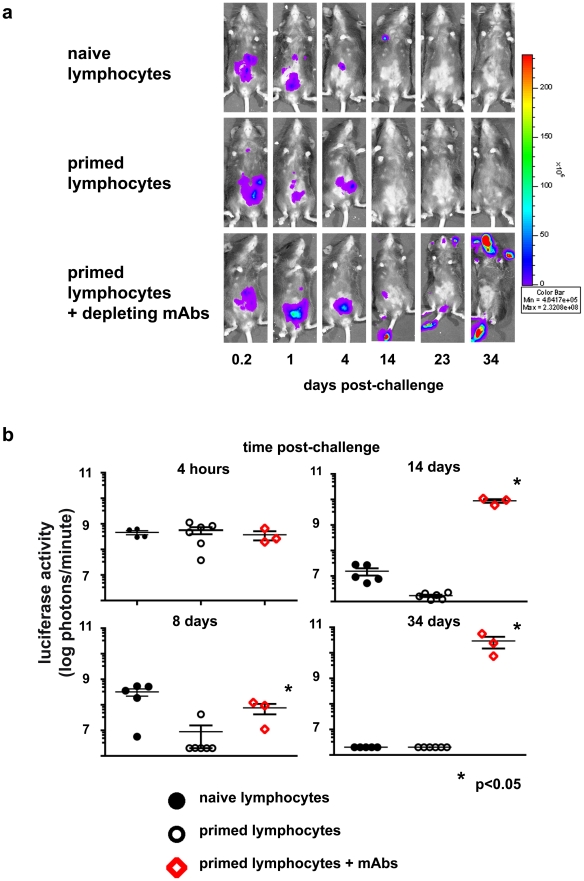
Protection conferred on naïve RAG1ko mice by adoptive transfer of primed lymphocytes is abrogated by simultaneous administration of T cell-depleting monoclonal antibody cocktail. Groups of RAG1^ko^ mice received 2×10^6^ NK-depleted hepatic lymphocytes from naïve or vaccinia virus-primed mice; a third group received both the primed lymphocytes and T cell-depleting monoclonal antibody cocktail simultaneously. All groups were challenged with 1×10^5^ pfu rVV-luc *ip* 5 days later. (a) IVIS images of representative mice from the indicated recipient groups are shown at various time points following challenge with rVV-luc. (b) The measurements of rVV-luc viral loads in each group over time are shown, with relative viral loads based on the number of photons emitted, normalized for a 1 minute exposure. The measurement for each individual mouse is plotted, and the mean is indicated by the centerline with error bars representing the SEM. Statistical analyses were performed using t tests and Mann-Whitney analysis comparing the primed lymphocyte transferred & T-depleted group and the isotype-treated groups at various time points to that of the naïve control group. Statistically significant differences between the depleted group and the isotype-treated groups post-challenge are indicated by asterisks (p<0.05). Results are representative of 3 independent experiments.

Our initial experiments in the IgH^ko^ model system indicated that a VV-primed Thy1^+^, non-T- non B- cell population was capable of providing host protection upon secondary exposure to the virus. However, despite the extreme efficiency of depletion (>99%; **Fig. 1a** and Fig. **2** **, and b**) achieved in the IgH^ko^ system, *in vivo* depletion via monoclonal antibody administration was not 100% efficient. Therefore, it remained a formal possibility that the protection we observed might be mediated by residual T cells. To address the possibility of residual T cell contamination, and confirm that a population of memory Thy1^+^ NK cells could confer protection against vaccinia virus, we evaluated the anti-viral activity of these cells in an adoptive transfer system. First, we wanted to establish that any protection we might observe in mice receiving these transferred cells was mediated by the transferred NK cell populations and not by contaminating T cells. To determine if we could adequately control for contaminating T lymphocytes in the transfers, we isolated NK cell-depleted liver mononuclear cell preparations from enzymatically-dissociated livers of naïve and vaccinia virus-primed mice by percoll gradient separation followed by magnetic separation of non-NK cells. We then transferred 2×10^6^ of these NK-depleted mononuclear cells (a mixture of T cells, B cells, macrophages, DCs, and granulocytes) into naïve RAG1^ko^ mice; one group received naïve NK-depleted cells, one group received VV-primed non-NK cells, and a third group received VV-primed non-NK cells in conjunction with the administration of the T cell-depleting monoclonal antibody cocktail utilized in the IgH^ko^ depletion experiments described above. Five days after the transfer of these cells, we challenged the recipients with 1×10^5^ pfu of rVV-luc ip and monitored their ability to control infection using the IVIS system. Mice that received naïve or VV-primed NK-depleted cells cleared the infection with kinetics consistent with a primary (naïve recipients) or secondary (primed recipients) T cell response (**Fig. 6**). However, the mice that received the T cell-depleting monoclonal antibody with the VV-primed non-NK cells were unable to control infection and had to be sacrificed at d34 post-challenge. These results indicate that even when transferring large numbers of primed T lymphocytes into naïve RAG1^ko^ hosts, administration of the T cell-depleting monoclonal antibody cocktail abrogated the protection afforded by the transferred cells.

We then isolated NK cells from the collagenase-treated livers of B6 or B6 congenic mice a minimum of 6 months after these mice had received either PBS (naïve) or 1×10^7^ pfu rVVluc (primed) ip. We utilized two congenic mouse models in these studies; B6.PL mice, which express the CD90.1 isoform of CD90, or B6.SJL mice, which express the CD45.1 isoform of CD45. The B6, IgH^ko^, and RAG^ko^ mouse models utilized in these studies express CD90.2 and CD45.2. Blood mononuclear cells were isolated from livers after enzymatic digestion followed by Percoll gradient centrifugation, and NK cells were isolated from this cell fraction using the MACS NK cell isolation kit and autoMACS instrument. Purified NK cells were then separated into Thy1^−^ and Thy1^+^ populations. Three distinct cell fractions were adoptively transferred into naïve RAG1^ko^ mice: 5×10^6^ NK-depleted cells (a mixture of T cells, B cells, macrophages, DCs, and granulocytes), 1×10^5^ Thy1^−^ NK cells, and 1×10^5^ Thy1^+^ NK cells. The groups of mice receiving the NK cell populations were treated with the T cell-depleting monoclonal antibody cocktail at the time of transfer to ensure that any contaminating T cells were eliminated. Thy1^+^ NK cells, but not T cells could be detected in the spleen or peripheral blood of RAG1^ko^ hosts that received purified liver Thy1^+^ NK cell transfers 4 weeks after transfer and challenge (**Fig. S3**); as expected, donor T cells were readily apparent in the peripheral blood of mice receiving NK-depleted liver cell transfers (**Fig. S3**), but were not detectable in mice receiving NK cell transfers.

**Figure 7 ppat-1002141-g007:**
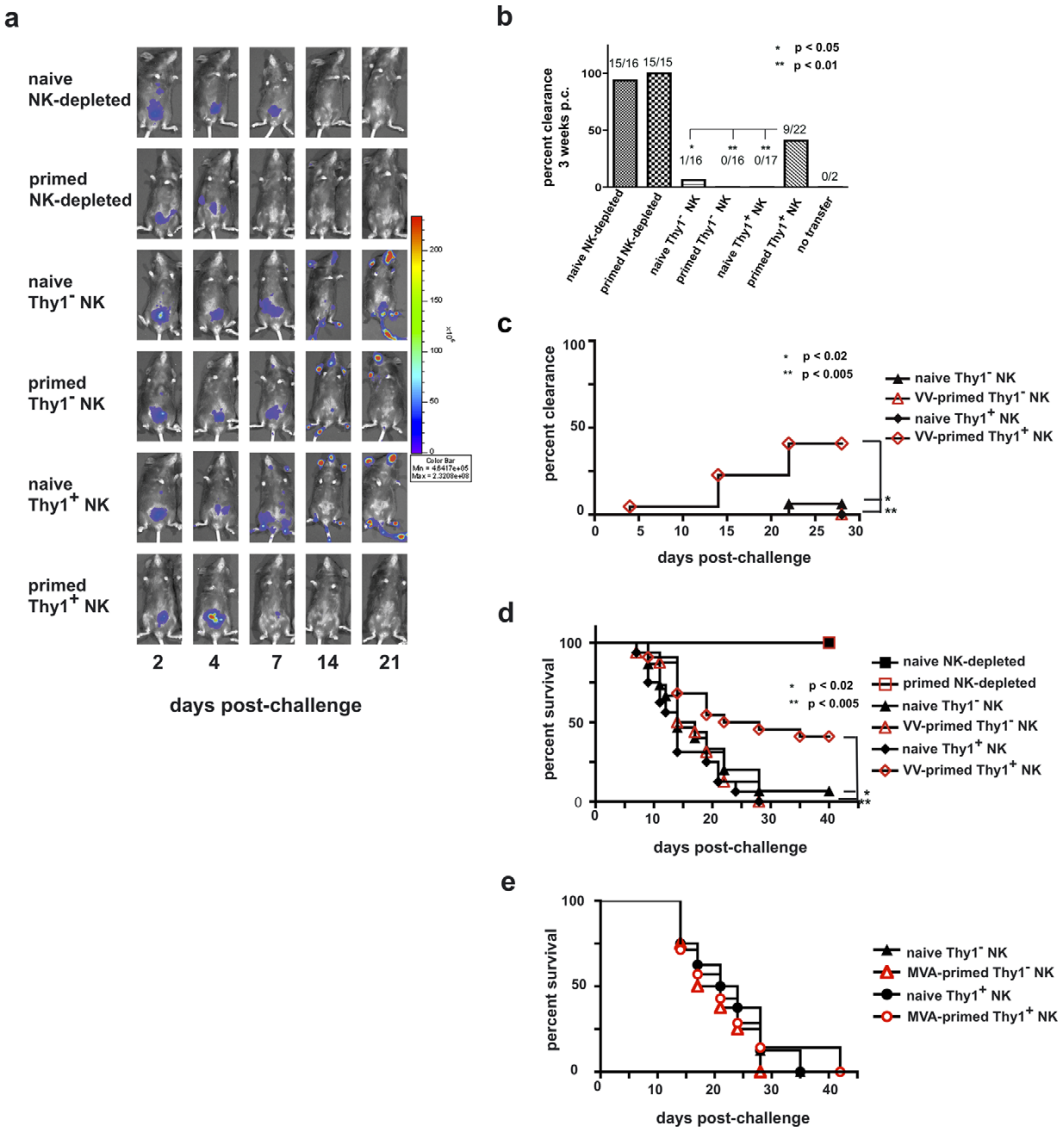
Innate immune protection against a vaccinia virus challenge is conferred on naïve RAG1^ko^ mice by the adoptive transfer of primed Thy1^+^ liver-resident NK cells. Six months after priming, 1×10^5^ Thy1^+^ liver NK cells, 1×10^5^ Thy1^−^ liver NK cells, or 5×10^6^ NK cell-depleted liver mononuclear cells from vaccinia virus-primed or naïve mice were adoptively transferred into RAG1^ko^ mice. At the same time, mice were administered either control immunoglobulins (those mice that received NK-depleted cell transfers) or T cell-depleting monoclonal antibodies (those mice that received Thy1^-^ and Thy1^+^ NK cell transfers). One week later the mice were challenged with 1×10^5^ pfu rVV-luc intraperitoneally, and viral loads were monitored by IVIS imaging. (**a**) IVIS images of representative mice from each group over time following challenge are shown. (**b**) The ability of RAG1^ko^ mice to resolve vaccinia virus infection by 3 weeks post-challenge is represented as the percent clearance within each group. Results were compiled from 4 separate experiments. RAG1^ko^ mice that received primed Thy1^+^ liver NK cells showed enhanced clearance of vaccinia virus as compared to mice that received naïve Thy1^+^ NK cells, naïve Thy1-depleted NK cells, and primed Thy1-depleted NK cells (two-tailed Fischer test between groups). All statistics compare the test group relative to the group that received primed Thy1^+^ liver-resident NK cells. (**c**) The ability of RAG1^ko^ mice to clear vaccinia virus infection throughout the course of challenge is represented as the percent clearance within each group. Results represent 4 separate experiments. RAG1^ko^ mice that received primed Thy1^+^ liver NK cells showed enhanced protection against vaccinia virus challenge as compared to mice that received naïve Thy1^+^ NK cells, naïve Thy1^−^ NK cells, and primed Thy1^−^ NK cells. Statistical analysis was performed to compare Kaplan-Meier survival curves using GraphPad Prism 4 software. All statistics compare the test group relative to the group that received primed Thy1^+^ liver-resident NK cells. Protection provided by transferred primed Thy1^+^ hepatic NK cells surpassed the protection observed in mice that received hepatic naïve Thy1^−^ NK cells (using the log rank test; p = 0.0168), primed Thy1^−^ NK cells (p = 0.0043), and naïve Thy1^+^ NK (p = 0.0033). (**d**) Kaplan-Meier curves showing the survival of naïve RAG1^ko^ recipients of the indicated adoptively transferred cell populations. Results represent the compilation of 4 separate experiments. RAG1^ko^ mice that received primed Thy1^+^ liver NK cells showed enhanced survival of vaccinia virus challenge as compared to mice that received naïve Thy1^+^ NK cells, naïve Thy1^−^ NK cells, or primed Thy1^−^ NK cells. Statistical analysis was performed as in (c), demonstrating that host protection provided by transferred primed Thy1^+^ hepatic NK cells surpassed the protection observed in mice that received hepatic naïve Thy1^−^ NK cells (log rank test; p = 0.013), primed Thy1^−^ NK cells (p = 0.0049), or naïve Thy1^+^ NK (p = 0.0004). (**e**) Kaplan-Meier curves showing the survival of naïve RAG1^ko^ recipients of the indicated adoptively transferred cell populations isolated from livers of naïve and MVA-primed mice. RAG1^ko^ mice that received MVA-primed Thy1^+^ liver NK cells showed no evidence of enhanced survival following rVV-luc challenge as compared to mice that received naïve Thy1^+^ NK cells, naïve Thy1^−^ NK cells, or MVA-primed Thy1^−^ NK cells. Statistical analysis was performed as in (c and d). Results shown are representative of 2 independent experimental replicates.

Five days after cell transfers (and concurrent T cell-depleting antibody administration for recipients of purified NK cell population transfers), we challenged these RAG1^ko^ mice with 1×10^5^ pfu rVV-luc ip and monitored the mice for viral burdens using the IVIS system. Representative IVIS images of individual mice from each of the experimental groups are shown in **Fig. 7a**, and the persistence or clearance of virus in each mouse from each group in 4 separate experiments are summarized in **Fig. 7** **, b and c**. As predicted, the RAG1^ko^ mice that received naïve, NK cell-depleted liver lymphocytes (15/16 mice; 94%) resolved the vaccinia virus challenge within 2 weeks, and the RAG1^ko^ mice that received the VV-primed NK cell-depleted liver lymphocytes (15/15; 100%) resolved the vaccinia virus challenge within 1 week (**Figs. 7b and c** **,** and data not shown). However, the groups of RAG1^ko^ mice that received naive Thy1^−^ (1/16; 6.25%) or Thy1^+^ (0/17; 0%) NK cell populations, or VV-primed Thy1^−^ (0/16; 0%) NK cells were unable to resolve vaccinia virus infections. Strikingly, a significant proportion of RAG1^ko^ mice that received VV-primed Thy1^+^ NK cell transfers (9/22; 41%) were able to clear the challenge vaccinia virus infection by three weeks after challenge, with viral clearance provided by transferred VV-primed Thy1^+^ NK cells significantly enhanced over mice receiving VV-primed Thy1^−^ NK cells (two-tailed t-test; p<0.01), naïve Thy1^−^NK cells (p<0.05), and naïve Thy1^+^ NK cells (p<0.01) transfers (**Fig. 7b and c**). Survival in these groups mirrored the clearance we observed by IVIS (**Fig. 7d**). All mice that received NK cell-depleted cells in transfer (cells that contained T lymphocytes) survived; however, of NK cell transfer recipients, only those that received the VV-primed Thy1^+^ NK cells were afforded significant protection, as shown by a survival rate of greater than 40% (9/22 recipients). We also wanted to determine if a highly attenuated poxvirus strain, MVA, was as capable of priming for protective NK cell memory as replication competent VV (Western Reserve strain). We transferred 1x10^5^ Thy1^−^ or Thy1^+^ NK cells isolated from the livers of naïve or MVA-primed RAG1^ko^ mice into naïve RAG1^ko^ recipients, then challenged the recipients with 1×10^5^ pfu rVV-luc IP 5 days after NK transfer. As shown in **Fig. 7e**, no significant protection against a replication-competent VV was observed in any of the recipients. These data indicate that VV-primed, but not MVA-primed, Thy1^+^ NK cells can clear a lethal systemic challenge with VV.

**Figure 8:**
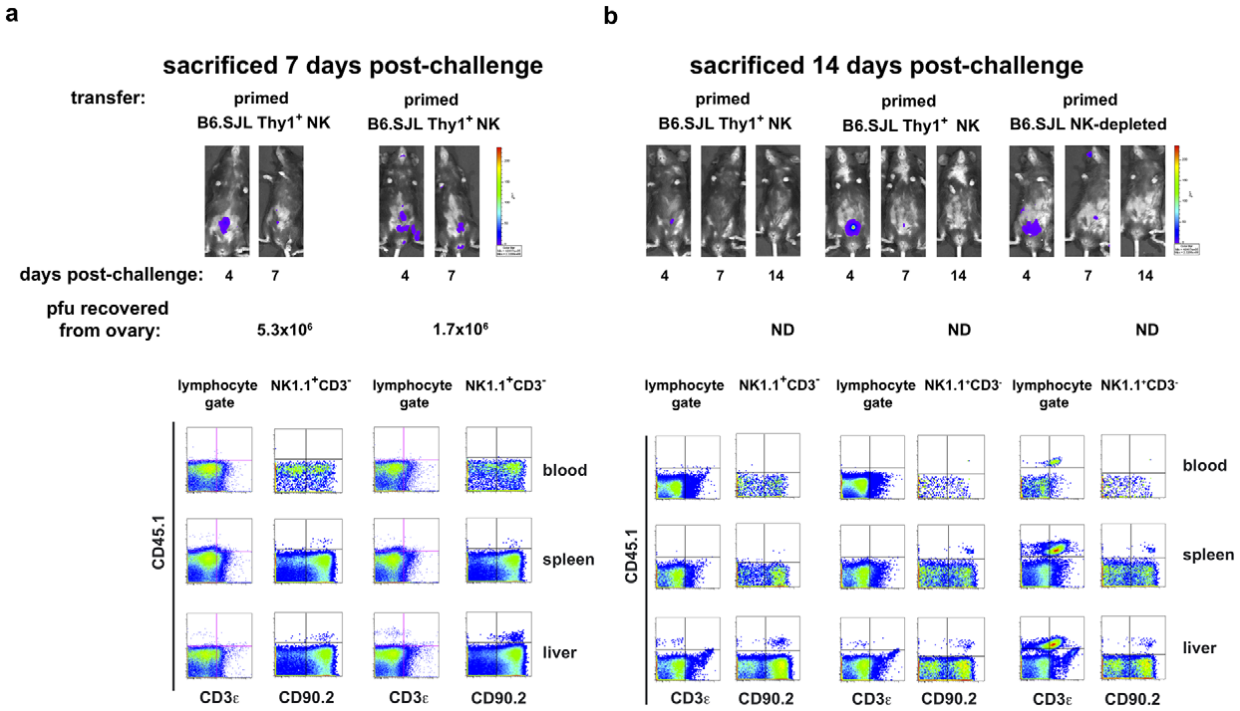
Protected RAG1^ko^ hosts contain adoptively transferred Thy1^+^ NK cells, not contaminating T lymphocytes. RAG1^ko^ mice (CD45.2^+^CD90.2^+^) received transfers of 2×10^6^ NK cell-depleted mononuclear cells, or 1×10^5^ Thy1(CD90)^+^ NK cells from livers of naïve or vaccinia virus-primed B6.SJL mice (CD45.1^+^CD90.2^+^). Coincident with the transfers, mice received *ip* a mixture of isotype control (NK-depleted recipients) or T cell-depleting (purified NK cell recipients) monoclonal antibodies. All recipients were challenged with 1×10^5^ pfu rVV-luc *ip* 5 days post-transfer. Shown are representative results from individual animals at 7 days (a) or 14 days (b) post-challenge. Mice were imaged via IVIS analysis through the course of the infection. At sacrifice, mononuclear cell suspensions were isolated from blood, spleens, and livers and analyzed via flow cytometry. Ovaries were harvested in 10 mM Tris buffer pH 9.0 and snap frozen in a dry ice–ethanol bath prior to processing and evaluation using a standard plaque formation assay on CV-1 cells. The recovered pfu/ovary are listed below each series of images (**ND** =  none detected). Results are representative of two independent experiments.

To verify that the protection observed in these studies is afforded by the transferred Thy1 (CD90)^+^ NK cells and not contaminating T lymphocytes, we isolated either NK-depleted liver lymphocytes (2×10^6^/transfer) or liver Thy1^+^NK cells (1×10^5^/transfer) from B6.SJL (CD45.1^+^CD90.2^+^) mice and transferred them into naïve RAG1^KO^ mice (CD45.2^+^CD90.2^+^) with simultaneous administration of the T depleting monoclonal antibody cocktail to the mice receiving the transferred NK cells. Five days after transfer, recipients were infected with 1×10^5^ pfu rVV-luc *ip* The course of the vaccinia virus infection in these animals is indicated by both IVIS images and classical pfu assay (**Fig. 8**). We were able to establish that the control (d7 post-challenge; **Fig. 8a**) and resolution (d14 post-challenge; **Fig. 8b**) of challenge vaccinia virus infection observed in animals receiving primed B6.SJL Thy1^+^ (CD45.1^+^CD90.2^+^) NK cell transfers occurred in the absence of contaminating T cells (CD45.1^+^CD3^+^) and in the presence of the transferred Thy1^+^ NK cells (CD3^−^NK1.1^+^CD45.1^+^CD90^+^). The protection and clearance of virus mediated *in vivo* by adoptively transferred B6.SJL NK-depleted liver lymphocytes (including T cells; CD3^+^CD45.1^+^CD90.2^+^) at d14 post-challenge is also shown (**Fig. 8b**). These results indicate that the protection observed against vaccinia virus challenges in recipients of primed Thy1^+^ NK cells is mediated by the transferred NK cells and is not a consequence of contaminating T lymphocytes in the transferred cells.

## Discussion

In these studies, we show that innate memory develops in response to a viral infection in the absence of an identified cognate receptor-virus interaction. The enhanced control of vaccinia virus in primed, T-depleted IgH^ko^ mice was apparent by 4 days post-challenge, indicating that the innate responses were both potent and rapid. That we observed this innate protection in animals that had resolved primary infection greater than 6 months prior to the secondary challenge has two important implications. First, it suggests that the protection represents a ‘memory’ response, rather than the persistence of a residual population of activated effectors. Second, the innate memory that formed following exposure to vaccinia virus is extremely durable. We further established that the complete protection we observed in the primed, T- depleted IgH^ko^ mice was abrogated with the addition of the Thy1-depleting antibody to the T cell depletion cocktail, indicating that innate memory to vaccinia virus resides within a Thy1^+^ non-T and non-B cell population. Perhaps the most striking result we observed in these experiments was that the memory provided by this innate Thy1^+^ cell population was manifested not only in enhanced control of vaccinia virus infection during the early stages of infection, but proved to be sufficiently potent to protect the infected hosts against a systemic lethal dose of virus in the absence of classical adaptive immune cells.

We performed experiments to determine if the memory Thy1^+^ NK cell that responded to vaccinia virus had a unique, persistent phenotype. *In vitro* stimulation assays established that the NK cells from primed animals responded more vigorously than NK cells from naïve animals, with increased CD69 upregulation and degranulation upon exposure to both priming antigens (plate-bound vaccinia virus) and a non- antigen-specific stimulus (plate-bound anti-NK1.1). These results indicate that primed hepatic NK cells develop and maintain an enhanced capacity for activation, not just enhanced pathogen-specific recall. These results also suggest that VV-specific memory NK cells may recognize subsequent exposure to vaccinia virus through a combination of signaling related to an induced self profile and direct recognition of virally-encoded proteins. That we observed only small differences in activation between NK cells stimulated with plate-bound vaccinia virus and the unrelated virus adenovirus suggests that the activation of NK cells is not driven predominantly by pathogen-encoded antigens, although it remains a formal possibility that formaldehyde fixation of the virus in these studies may have diminished the capacity of NK cells to interact directly with and recognize immunogenic portions of the virus.

We further sought to define a durable cell surface marker expression profile acquired after a primary infection that might identify a memory cell subpopulation within the liver-resident Thy1^+^ NK cell compartment. We observed significant increases in total NK cell numbers in spleen (data not shown) and liver, consistent with previous reports of global expansions of NK cell populations after viral infections[7]. We also observed a preferential expansion of the Thy1^+^ NK cell population, as determined both by the percentage and the absolute numbers of Thy1^+^ NK cells. Our EdU incorporation experiments (**Fig. 5**) established that the expansion in total NK cell numbers, and Thy1^+^ NK cells in particular, is driven by *in situ* proliferation of Thy1^+^ NK cells during the first week of infection, not the accumulation of effector cells migrating from peripheral sites. This expansion of Thy1^+^ NK cells was accompanied by a preferential increase in the representation of a KLRG1^+^ subset of cells that was predominantly CD27^lo^CD11b^hi^Ly6c^hi^(**Fig. 4**, **b** and **c**), a profile consistent with these cells being mature, activated NK cell effectors[24], [25]. Importantly, in accordance with an earlier report[7], we observed no preferential expansion of the Ly49H^+^ NK cell subset that mediates innate memory against MCMV[2]. These data suggest that innate memory to vaccinia virus is mediated by a subset of NK cells that is distinct from those that form innate memory to MCMV. However, by 4 months post-challenge, the phenotypic differences between the Thy1^+^ liver NK cell population in control and vaccinia virus-infected mice observed during the acute phase of infection was no longer apparent. This observation is consistent with a recent report that previously stimulated NK cells retain enhanced functional capacity in the absence of a phenotypic profile that distinguishes these cells from naïve NK cells[26].

A significant concern that must be addressed when assessing the protection against infection described in the present studies is whether any residual functional T cell populations might be present that would be capable of mediating the protection. In the IgH^ko^ system, we were able to achieve extremely efficient depletion of T cells as measured by anti-CD3 staining (approximately 99% at 1 week post-challenge). However, while this T cell depletion in the naïve and primed IgHko groups of mice was efficient, complete depletion of all T cells was not possible. Despite the fact that T cell depletion completely abrogated protection in naïve IgH^ko^ recipients, it therefore remained a formal possibility that residual T cells in the primed mice could contribute to host protection. We therefore addressed this issue by adoptively transferring highly purified NK cell subsets into naïve RAG1^ko^ hosts and concurrently administering the same T cell-depleting cocktail (**Figs. 6** **-** [Fig ppat-1002141-g007] **8**; **Fig. S3**).

We were able to demonstrate that protection against vaccinia virus could be transmitted to naïve RAG1^ko^ hosts by adoptive transfer of purified vaccinia virus-primed Thy1^+^ liver NK cells. Of the naive RAG1^ko^ mice that received 1×10^5^ primed Thy1^+^ liver NK cells, 41% (9/22) were able to resolve a systemic, lethal vaccinia virus challenge infection, while only 2% (1/49) of RAG1^ko^ mice that received other purified NK cell subpopulations were able to resolve and survive infection **(** **Fig. 7** **)**. Our inability to achieve protection in 100% of RAG1^ko^ mice by transfer of primed Thy1^+^ liver NK cells may be a consequence of limiting numbers of Thy1^+^ liver NK cells and the inefficiency associated with the cell transfers.

We administered the T cell-depleting monoclonal antibody cocktail to NK cell transfer recipients at the time of transfer to prevent contaminating T cells from contributing to the protection of these recipients. Indeed, as shown in **Fig. 6**, even when transferring 20-fold more cells —cell preparations that contained a significant proportion of primed T cells, in contrast to the highly purified NK cell populations transferred in these experiments — we observed that the T cell depleting cocktail abolished protection (as measured by both IVIS and survival). Further, when we looked for the presence of contaminating T cells at d7, d14, and d28 post-challenge (d12, d19, and d33 post-transfer and antibody administration, respectively), we were able to identify transferred Thy1^+^ NK cells but were unable to detect T cells in the peripheral blood, livers, or spleens of NK cell transfer recipients (**Fig.8**; **Fig. S3**). These data indicate that the protection we observed in mice that received VV-primed Thy1^+^ liver NK cell transfers was indeed conferred by this population of cells (**Figs. 7** **and** **8** **)**.

We also investigated whether MVA-primed Thy1^+^ NK cells from RAG1^ko^ hosts were capable of conferring the same protection upon transfer into naïve immunodeficient hosts and determined that priming with the highly attenuated MVA virus was not sufficiently robust to generate protective memory comparable to that induced by priming with live, replication competent VV. There are several possible explanations for why MVA priming did not engender the same degree of protective innate memory. One possibility is that the viral product(s) necessary for NK memory recognition of VV were lost in the attenuation process. A recent study [3] was able to demonstrate antigen-specific memory NK cells induced by a range of virus-like particles (VLPs), including VLPs containing antigens derived from pathogens not endemic to the mouse population (like HIV-1), suggesting that this mechanism is unlikely. Even the highly attenuated MVA strain has a rather large genome encoding an extremely diverse range of potential antigens, including a wide range of antigens shared with the vaccinia virus strain used in the challenges. A second possibility is that NK memory formation to VV requires conditioning from adaptive cell populations at some point during the priming response. However, the ability of RAG1^ko^ hosts to generate protective, pathogen-specific NK memory upon exposure to a variety of VLPs also suggests that the presence of classical adaptive lymphocytes are not necessary during the priming phase for effective innate memory formation. A third possibility is that, as has been observed for classical memory [27], innate memory is generated more potently in response to a live, replication-competent agent. If an ‘active’ infection stimulates a more robust response, it may do so is by stimulating enhanced expression of self-molecules in cells under the stress of active infection and replication. In such a scenario, NK cells might well represent the population best equipped to respond to those stressed-self indicators through their array of germline-encoded receptors.

These data demonstrate that VV infection generates a liver-resident Thy1^+^ NK cell population capable of mediating a protective innate memory response. It is not clear how primed Thy1^+^ liver NK cells provide this protection against VV infection. It will be important to determine whether innate memory NK cells respond to pathogens more effectively than naïve memory NK cells because they have enhanced lytic capacity, increased stores of premade effector molecules (such as cytokines, granzyme, and/or perforin), superior proliferative capacity, or an as yet undefined property that facilitates a particularly robust effector response. While the *in vitro* stimulation assay shown in this study indicates that the primed NK cells do possess enhanced effector capacity (**Fig. 3**), the precise mechanism by which this is mediated remains unclear.

The mechanism by which primed Thy1^+^ liver NK cells recognize vaccinia virus upon secondary exposure is of great importance. There are several hypotheses, not mutually exclusive, that could account for pathogen-specific recognition of a previously encountered pathogen. First, there may exist a whole class of previously unidentified receptors expressed by innate cell populations that, like Ly49H, have co-evolved to specifically recognize the various pathogens endemic to the host population. Such molecules could act as highly pathogen-specific pattern recognition PRRs. Indeed, the murine homologue of the human NKp46 receptor (also known as Ncr1) is critical for host defense against influenza[28], and human NKp46 has been shown to bind viral hemagglutinins[29], [30], [31]. Moreover, previous studies have shown that genes within the NK complex on chromosome 6 in B6 mice contribute to NK cell-mediated immune responses to ectromelia[32], [33], an orthopoxvirus closely related to vaccinia virus. This finding suggests that an as-yet-unidentified receptor capable of recognizing a poxviral product may be encoded within this region.

A second potential mechanism by which pathogen-specific recognition could be mediated through germline-encoded receptors could reflect alterations in the ability of NK receptors to interact with MHC class I and MHC class I-like ligands when binding and/or presenting viral epitopes. Alternatively, infection by pathogen could induce a state within the cell that results in the display of an ‘infected-self’- or ‘stressed-self’- associated profile of protein expression that can then be recognized by receptors on innate memory NK cells. One consequence of such an ‘infected-self’ mechanism of recognition would be that the innate memory formed in response to one pathogen might provide protection against challenge with heterologous pathogens that induce a similar molecular self-profile in infected cells. The phenomenon of NK cell recognition and response to induced-self molecules has previously been implicated in both infections and oncogenesis[34], [35], [36], [37], where ligands MULT1[38], H60[39], and Rae-1[39], [40]) for the activating receptor NKG2D are preferentially induced upon infection or cellular transformation[36], [37], [41]. Indeed, activation through NKG2D has been established as a critical component of the NK cell response to ectromelia virus infection[42]. That protective NK cell responses to poxvirus infections have been tied to both an as-yet unidentified gene in the B6 NK receptor complex[32], [33] and to NKG2D activity[42] suggests that both the pathogen-specific PRR and ‘infected-self’ mechanisms may contribute to NK cell responses to poxviruses. Indeed, our *in vitro* assays showing enhanced responsiveness of vaccinia virus-primed NK cells to stimulation through the NK1.1 receptor pathway as well as direct exposure to vaccinia virus would be consistent with both mechanisms playing a significant role in NK memory cell recognition of poxviruses.

These studies have established a model of innate memory to a pathogen in a system in which there is no known pathogen-specific receptor expressed by innate cell populations. This system will allow us to explore models of innate memory recognition further. Understanding the nature of that recognition will be critical for harnessing innate memory for prophylactic or therapeutic use. The stimulation of innate memory targeted to specific pathogens might represent a novel and powerful approach for the design of future vaccination strategies.

## Materials and Methods

### Ethics statement

This study was carried out in strict accordance with the recommendations in the Guide for the Care and Use of Laboratory Animals of the National Institutes of Health. All animals are treated humanely and in accordance with the policies of the Beth Israel Deaconess Medical Center (BIDMC), the regulations of the Animal Welfare Act, and other laws and policies of the federal government and other agencies. All mice were maintained under specific-pathogen-free conditions and research on mice was approved by the BIDMC Institutional Animal Care and Use Committee (IACUC) under protocol #095-2009. All efforts were made to minimize suffering of animals in this study.

### Mice

Age-matched adult female C57Bl/6, B6.PL (Thy1.1 congenic), B6.SJL (CD45.1 congenic), IgHμ^ko^ (μMT[13], [14]), and RAG1^ko^ were obtained from Jackson Laboratories (Bar Harbor, ME). All mice were maintained in the Harvard Institute of Medicine and Beth Israel Deaconess Medical Center (BIDMC) Animal Research facilities and used in accordance with protocols approved by the Institutional Animal Care and Use Committees of BIDMC, Harvard Institutes of Medicine, and Harvard Medical School.

### Viruses

Stocks of recombinant vaccinia were prepared as previously described[43]. Briefly, seed stocks of recombinant vaccinia virus (Western Reserve strain) expressing firefly luciferase[44] (rVV-luc; kindly provided by Michael Seaman (BIDMC; Boston, MA USA) were added to HeLa cell monolayers at an MOI of 1. Seeded monolayers were harvested and washed into 10 mM Tris pH 9.0 48–72 hours after seeding, and cell-associated virus was released from cells by 3 freeze-thaw-sonication cycles followed by centrifugation at 850 g for 10 min at 4°C. Supernatants (containing free virus) were then layered over a 36% sucrose cushion and centrifuged for 2 hours at 27,000 rpm (1.33×10^5^ g) at 4°C. After centrifugation, pellets containing purified cell-free virus were resuspended in 10 mM Tris pH 9.0, aliquoted, and titered using a standard *in vitr*o plaque assay using CV-1 cells[43]. Recombinant Modified Vaccinia Ankara virus expressing firefly luciferase [27] (rMVA-luc; were provided by Michael Seaman (BIDMC; Boston, MA USA).

### Antibodies for *in vivo* cell depletion

Sterile, certified low endotoxin preparations of monoclonal antibodies H57–597 (hamster IgG anti-mouse TCRβ)[16], UC7-13D5 (hamster IgG3 anti-mouse TCRγδ)[17], 30H12 (rat IgG2b anti-mouse Thy1.2)[23], GK1.5 (rat IgG2b anti-mouse CD4)[15], LTF-2 (rat IgG2b anti-Keyhole Limpet Hemagglutinin (KLH); used as an isotype control), as well as a polyclonal preparation of purified low endotoxin hamster IgG, were purchased from Bio-X-cell cell culture services (West Lebanon, NH), diluted in sterile PBS to the desired concentration, and administered intraperitoneally (ip). Depleting or isotype control antibodies were administered intraperitoneally to groups of naïve and VV-primed groups of IgH^ko^ mice every 2 weeks starting at least 1 week prior to secondary challenge. For adoptive transfer experiments, appropriate depleting or isotype control antibodies were administered intraperitoneally concurrently with transfer of the indicated cell populations.

### Antibodies and secondary reagents for flow cytometric analyses and cell sorting

Fluorescently conjugated antibodies specific for mouse CD3ε (clones 145-2C11, 17A2, and/or 500A2), CD11b (clone M1/70), CD16/32 (clone 24G2; for blocking Fc receptor-mediated binding of fluorescent labeled antibodies), CD49b (clone DX5), CD27 (clone LG.7F9), CD43 (clone eBioR2/60), CD45.1 (clone A20), CD45.2 (clone 104), CD62L (clone MEL-14), CD69 (clone H1.2F3), CD90.1 (clone HIS51), CD90.2 (clone 53-2.1), CD94 (clone 18d3), CD107a (clone 1D4B), CD107b (clone ABL-93), CD127 (clone A7R34), CD244.2 (B6 2b4 alloantigen; clone ebio244F4), KLRG1/MAFA (clone 2F1), Ly6c (clone AL-21) Ly49C/I/F/H (clone 14B11), Ly49H (clone 3D10), NKG2D (clone CX5), NK1.1 (clone PK136), TCRß (clone H57–597), and TCRγδ (clone GL-3) were obtained from either BD Biosciences (San Jose, CA USA) or eBioscience, Inc. (San Diego, CA USA).

### Isolation of cell populations

Livers were removed from naïve and vaccinia virus-primed B6, B6.SJL (CD45.2 congenic), or B6.PL (CD90.1 congenic) mice and diced in calcium-magnesium- free HBSS + 2% FBS prior to enzymatic dissociation. Diced livers were resuspended in a digestion buffer consisting of 500 µg/ml Collagenase D (>0.15 U/mg; Roche Applied Science) and 10 µg/ml DNAse I (2500 U/mg; Roche Applied Science) in HBSS + 2% FBS and agitated for 1 hour at 37°C. The resulting cell suspension was passed through a 70 micron filter and added to the cell suspensions released by dicing the livers harvested prior to digestion. The remaining pieces were washed two additional times, and the resulting cell suspensions were passed through a 70 micron filter and added to the previously pooled cell suspensions. The pooled cell suspensions were then spun at 500 g for 10 minutes at 20°C. The supernatant was discarded and pelleted cells were resuspended and washed 2 more times in HBSS + 2% FBS. The pelleted cells were then resuspended in 40% Percoll in 1x HBSS, then underlaid with an equal volume of 67% Percoll in 1x HBSS prior to centrifugation at 900 g for 30 minutes at 20°C. Cells at the interface between the two Percoll layers were saved and washed twice in MACS buffer (PBS +0.5% BSA +2.5 mM EDTA). Cells for phenotypic analysis were counted using Guava ExpressPlus software on a Guava easyCyte instrument (Millipore; Billerica, MA) prior to staining with fluorescently-conjugated monoclonal antibodies for analysis on an LSR II instrument in our laboratory. For preparation of adoptive cell transfers, two consecutive rounds of purification were performed: first the mouse NK cell isolation kit (cat # 130-090-864) was used in accordance with the manufacturer's protocols (miltenyi biotec; Bergisch Gladbach, Germany) to deplete non-NK cells on an autoMACS instrument. The enriched NK population from B6 or B6.SJL mice was then washed and separated via the AutoMACS (program ‘possel’; non-labeled fraction is Thy1^−^ NK, labeled fraction is Thy1^+^ NK) using MACS beads specific for CD90.2 (cat # 130-049-101, miltenyi biotec). The enriched NK population from B6.PL (CD90.1^+^CD90.2^−^) livers was incubated with antibodies specific for CD3ε, NK1.1, and Thy1.1 prior to flow cytometric sorting into Thy1^−^ NK (Thy1^−^CD3^−^NK1.1^+^) and Thy1^+^ (Thy1^+^CD3^−^NK1.1^+^) populations on a BD Vantage instrument in our laboratory.

Purified populations of NK cell-depleted blood mononuclear cells (control; 2–5×10^6^ cells/mouse), Thy1^−^ (1×10^5^ cells/mouse), or Thy1^+^ NK cells (1×10^5^ cells/mouse) in 1x PBS from naïve and vaccinia virus-primed mice, or naïve RAG1^ko^ and MVA-primed RAG1^ko^ mice, were transferred into age-matched female RAG1^ko^ recipients via tail vein injection using a 25 G needle. Within 5 days post-transfer, recipients were challenged with 1×10^5^ pfu of rVV-luc ip and monitored over time using the IVIS Illumina-II imaging system (Xenogen, inc.; Alameda, CA USA).

### *In vivo* monitoring of viral burdens

At numerous time points following challenge, rVV-luc burdens were monitored *in vivo* using the IVIS imaging system. Briefly, rVV-luc –infected and/or uninfected control animals were injected ip with 100 µl of a 30 mg/ml solution of firefly luciferin-D (Caliper Life Sciences; Hopkinton, MA, USA) in PBS, and 100 µl of a 20 mg/ml ketamine and 1.72 mg/ml xylazine mixture and imaged 14–16 minutes later in the IVIS series 100 imager (IgH^ko^ experiments) or IVIS- lumina II instrument (adoptive transfer experiments). Typical exposure times were 60 seconds (IVIS 100 series) or 30 seconds (IVIS-Lumina II); however, when higher viral burdens were present exposure times were shortened to ensure that images were not oversaturated, and all measurements were normalized for one minute exposure. Overlay images and luminescence measurements were made using Living Image software (version 2.50.1; Xenogen).

### *In vitro* stimulation assay

Tissue culture plates were treated with either carbonate binding buffer alone (unstimulated and PMA/ionomycin wells), 1 mg/ml control Ig (clone CI.8; Bio-X-cell) in binding buffer, 1 mg/ml anti-NK1.1 (clone PK-136; Bio-X-cell), 2×10^6^ pfu/ml vaccinia virus, or 2×10^6^ vp adenovirus in binding buffer overnight at 4°C. Wells were washed three times with HBSS + 2% FBS. Wells coated with vaccinia virus or adenovirus were treated with 2% formaldehyde in PBS for 20 minutes at room temperature, washed 4 additional times with complete DMEM + 10% FBS, and all wells were blocked for 2 hr at 4°C with cDMEM + 10% FBS. 5×10^5^ liver mononuclear cell preparations from groups of 8–12 mice were incubated in DMEM alone (unstimulated, antibody coated, and vaccinia coated wells) or DMEM with PMA and ionomycin (1 µg/ml and 5 µg/ml respectively) for 6 hours at 37°C. Cell suspensions were then recovered from the plate and processed for flow cytometric analysis.

### *In vivo* proliferation assay

Groups of B6 mice were infected with 1×10^7^ pfu rVV *ip* 12 hours prior to sacrifice, infected mice and a matching group of naïve mice were administered 250 µg of the thymidine analog 5-ethynyl-2′-deoxyuridine (EdU) in PBS *ip*. At sacrifice, mononuclear cells were isolated from livers and spleens, stained with monoclonal antibodies to NK1.1, CD3ε, CD90.2, and fixed. Fixed cells were then washed in a permeabilization buffer containing saponin, treated with Click-IT EdU staining buffer containing Alexa 647 azide to stain incorporated EdU, washed, and analyzed by flow cytometry.

### Vaccinia CV-1 plaque assay

Ovaries were harvested in 10 mM Tris pH 9.0 and snap frozen on a dry ice-ethanol bath. Samples were then thawed, homogenized, and snap frozen again. The samples were then thawed, vortexed, and serially diluted stepwise 1∶10 in duplicate in cDMEM + 10% FCS. 500 µl of each dilution was placed on a confluent layer of CV-1 cells in 6 well plates and incubated at 37°C for one hour, prior to aspiration and addition of 3 ml cDMEM + 10%FCS to each well. Plates were incubated for an additional 48 hours at 37°C prior to aspiration and staining and fixing with 500 µl of 0.1% crystal violet in 20% ethanol for 5 min. After removing the staining solution, the plates were air-dried and then counted. Viral loads in the ovaries were calculated based on the number of plaques, the dilution factor, and the volume of homogenate.

## Supporting Information

Figure S1 **Representative IgH^ko^ priming data.** Adult IgH^ko^ mice received 1×10^7^ pfu rVV-luc intraperitoneally and were monitored over time by IVIS imaging. Mice were rested a minimum of 6 months after initial clearance of the rVV-luc prior to use in challenge experiments.
(TIF)

Figure S2 **Representative IVIS imaging and ovarian pfu recovered from vaccinia virus-challenged IgHko mice.** IVIS imaging is shown of the viral load and distribution in individual IgHko mice throughout the course of the experiment. Unless otherwise noted, mice were sacrificed approximately 5 weeks after challenge. At the time of sacrifice, ovaries were harvested in 10 mM Tris buffer pH 9.0 and snap frozen in a dry ice–ethanol bath prior to processing and evaluation using a standard plaque formation assay on CV-1 cells. The recovered pfu/ovary are listed to the right of each series of images (**ND** =  none detected).
(TIF)

Figure S3 **NK cells transferred into RAG1^ko^ hosts persist and do not contain contaminating T lymphocytes at 4 weeks post-challenge.** RAG1^ko^ mice received transfers of 5×10^6^ NK cell-depleted mononuclear cells, 1×10^5^ Thy1^−^ NK cells, or 1×10^5^ Thy1^+^ NK cells from livers of naïve or vaccinia virus-primed wild type mice. Coincident with the transfers, mice received *ip* a mixture of isotype control (NK-depleted recipients) or T cell-depleting (purified NK cell recipients) monoclonal antibodies. All recipients were challenged with 1×10^5^ pfu rVV-luc *ip* 5 days post-transfer. **(a)** Shown are representative flow cytometric analyses of viable mononuclear cells from the peripheral blood (all recipients) and spleens (NK cell recipients only) 28 days post-challenge. **(b)** Flow cytometric plots of cell populations in RAG1ko hosts (CD90.2^+^) that received adoptive transfers of vaccinia virus-primed B6.PL Thy1(CD90.1)^+^ NK cells 4 weeks post-challenge by staining cell suspensions from livers and spleens For CD3, NK1.1, CD90.1, and CD90.2. The left panels are gated on total lymphocytes and show the absence of contaminating T cells (CD3^+^CD90.1^+^). Middle panels are gated on total lymphocytes and show the NK cell (CD3^−^NK1.1^+^) population and corresponding NK cell gate. The right panel are plots showing events within the NK cell gate shown in the middle panel, and show the presence of transferred Thy1(CD90)^+^ B6.PL NK cells [CD3^−^NK1.1^+^CD90.1^+^] within the host NK cell population (host Thy1(CD90)^+^NK are CD90.2^+^).
(TIF)
