# Correction: Thy1^+^ Nk Cells from Vaccinia Virus-Primed Mice Confer Protection against Vaccinia Virus Challenge in the Absence of Adaptive Lymphocytes

**DOI:** 10.1371/annotation/b29086ef-e08d-444c-8113-18a6dd429a7c

**Published:** 2011-08-18

**Authors:** Geoffrey O. Gillard, Maytal Bivas-Benita, Avi-Hai Hovav, Lauren E. Grandpre, Michael W. Panas, Michael S. Seaman, Barton F. Haynes, Norman L. Letvin

An error was introduced in the preparation of this article for publication. In the title, 'Thy1+ Nk Cells' should have an uppercase 'K'. The correct title should read: Thy1+ NK Cells from Vaccinia Virus-Primed Mice Confer Protection against Vaccinia Virus Challenge in the Absence of Adaptive Lymphocytes. The correct citation is: Gillard GO, Bivas-Benita M, Hovav A-H, Grandpre LE, Panas MW, et al. (2011) Thy1+ NK Cells from Vaccinia Virus-Primed Mice Confer Protection against Vaccinia Virus Challenge in the Absence of Adaptive Lymphocytes. PLoS Pathog 7(8): e1002141. doi:10.1371/journal.ppat.1002141

