# Supplementary figures and images for: Thy1+ Nk Cells from Vaccinia Virus-Primed Mice Confer Protection against Vaccinia Virus Challenge in the Absence of Adaptive Lymphocytes
Source: PLoS Pathog. 2011 Aug 4;7(8):e1002141. doi: 10.1371/journal.ppat.1002141 (PMC3150274; doi:10.1371/journal.ppat.1002141)

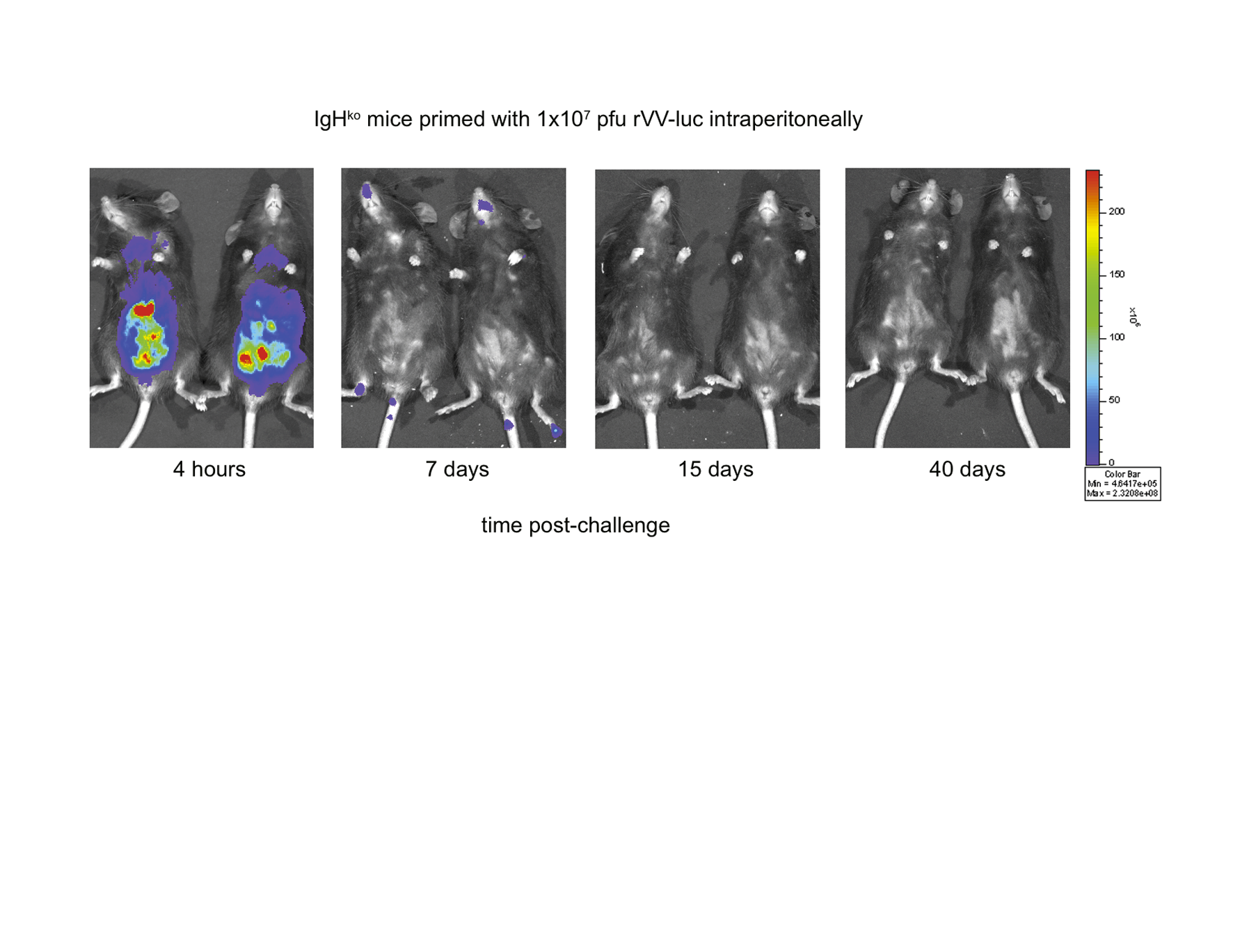

Supplement: Figure S1 — Representative IgHko priming data. Adult IgHko mice received 1×10^7^ pfu rVV-luc intraperitoneally and were monitored over time by IVIS imaging. Mice were rested a minimum of 6 months after initial clearance of the rVV-luc prior to use in challenge experiments. (TIF) [file ppat.1002141.s001.tif]

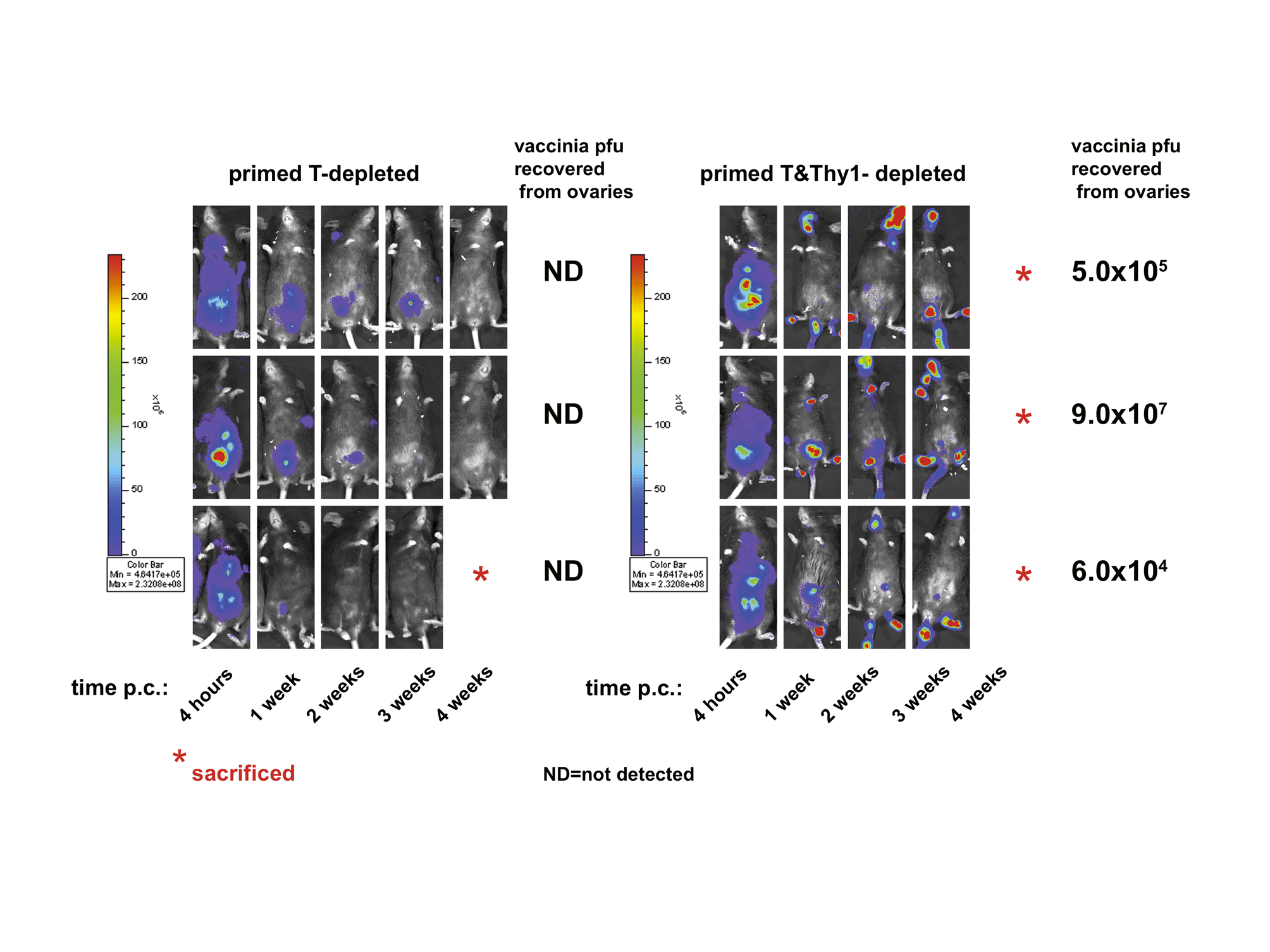

Supplement: Figure S2 — Representative IVIS imaging and ovarian pfu recovered from vaccinia virus-challenged IgHko mice. IVIS imaging is shown of the viral load and distribution in individual IgHko mice throughout the course of the experiment. Unless otherwise noted, mice were sacrificed approximately 5 weeks after challenge. At the time of sacrifice, ovaries were harvested in 10 mM Tris buffer pH 9.0 and snap frozen in a dry ice–ethanol bath prior to processing and evaluation using a standard plaque formation assay on CV-1 cells. The recovered pfu/ovary are listed to the right of each series of images (ND = none detected). (TIF) [file ppat.1002141.s002.tif]

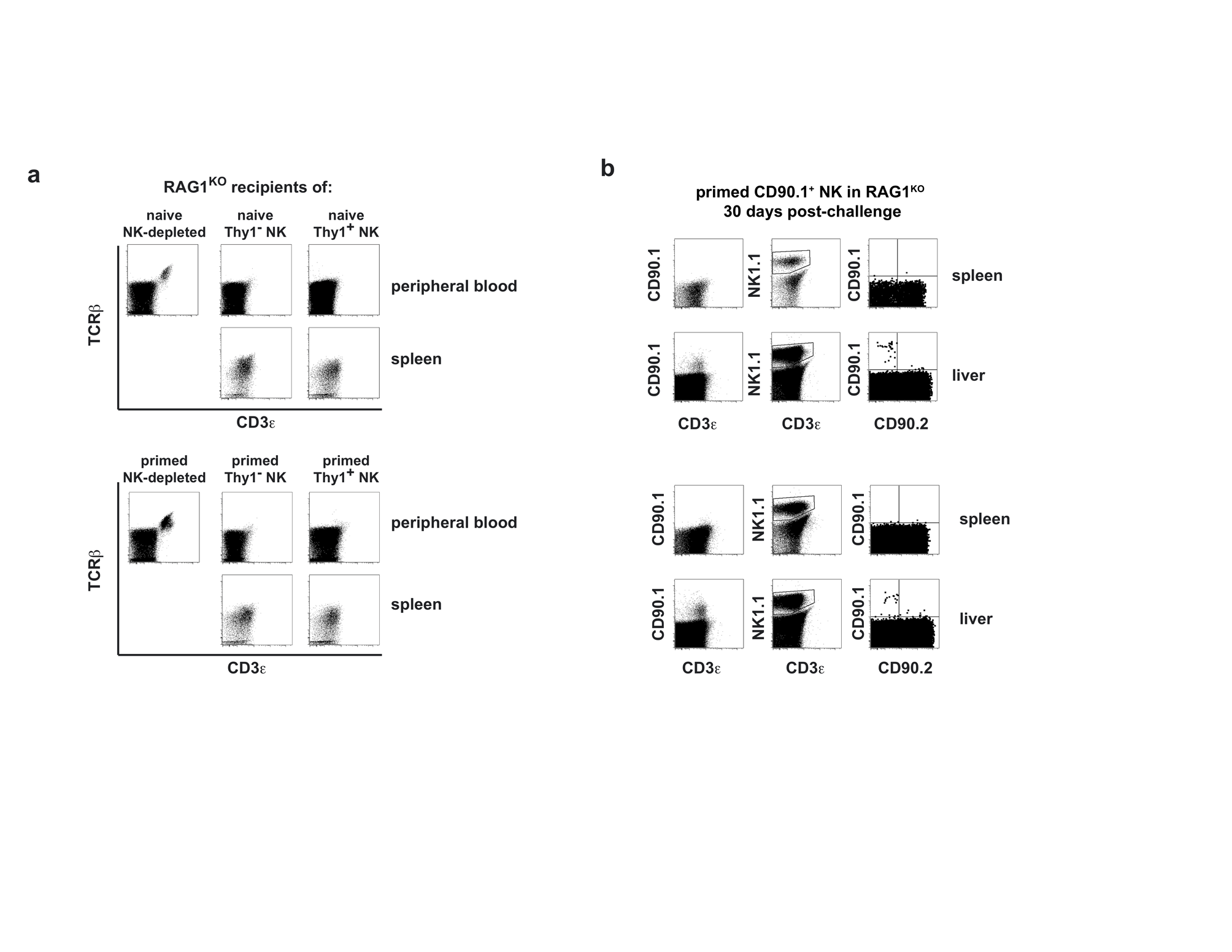

Supplement: Figure S3 — NK cells transferred into RAG1ko hosts persist and do not contain contaminating T lymphocytes at 4 weeks post-challenge. RAG1ko mice received transfers of 5×10^6^ NK cell-depleted mononuclear cells, 1×10^5^ Thy1− NK cells, or 1×10^5^ Thy1+ NK cells from livers of naïve or vaccinia virus-primed wild type mice. Coincident with the transfers, mice received ip a mixture of isotype control (NK-depleted recipients) or T cell-depleting (purified NK cell recipients) monoclonal antibodies. All recipients were challenged with 1×10^5^ pfu rVV-luc ip 5 days post-transfer. (a) Shown are representative flow cytometric analyses of viable mononuclear cells from the peripheral blood (all recipients) and spleens (NK cell recipients only) 28 days post-challenge. (b) Flow cytometric plots of cell populations in RAG1ko hosts (CD90.2+) that received adoptive transfers of vaccinia virus-primed B6.PL Thy1(CD90.1)+ NK cells 4 weeks post-challenge by staining cell suspensions from livers and spleens For CD3, NK1.1, CD90.1, and CD90.2. The left panels are gated on total lymphocytes and show the absence of contaminating T cells (CD3+CD90.1+). Middle panels are gated on total lymphocytes and show the NK cell (CD3−NK1.1+) population and corresponding NK cell gate. The right panel are plots showing events within the NK cell gate shown in the middle panel, and show the presence of transferred Thy1(CD90)+ B6.PL NK cells [CD3−NK1.1+CD90.1+] within the host NK cell population (host Thy1(CD90)+NK are CD90.2+). (TIF) [file ppat.1002141.s003.tif]
